# Improving the Care of Older Patients by Decreasing Potentially Inappropriate Medications, Potential Medication Omissions, and Serious Drug Events Using Pharmacogenomic Data about Variability in Metabolizing Many Medications by Seniors

**DOI:** 10.3390/geriatrics5040064

**Published:** 2020-09-27

**Authors:** Roger E. Thomas

**Affiliations:** Department of Family Medicine, Cumming School of Medicine, University of Calgary, Calgary, AB T2N4N1, Canada; rthomas@ucalgary.ca; Tel.: +1-403-220-0157

**Keywords:** seniors, reducing potentially inappropriate medications, potential medication omissions, serious drug events, pharmacogenomics, P450 cytochrome isoforms

## Abstract

Polypharmacy, potentially inappropriate medications (PIMs) identified by the American Geriatrics Society and Screening Tool of Older People’s Prescriptions (STOPP), potential prescribing omissions (PPOs) identified by Screening Tool to Alert to Right Treatment (START) and serious drug events (SDEs), are major problems for seniors. They correlate with increased risks of rehospitalization and death within six months of hospital discharge. About 75% of commonly prescribed medications are metabolized by P450 cytochrome enzymes. Electronic medical records (EMRs) providing integrated comprehensive pharmacogenomic advice are available only in very large health organizations. The study design of this article is a cross-sectional analysis of the American Geriatrics Society (AGS) and STOPP PIM and START PPO databases integrated with three P450 cytochrome enzyme databases (Flockhart Tables, DrugBank, and Rx Files) and the data are reported using the Strengthening the Reporting of Observational Studies in Epidemiology (STROBE) Statement: guidelines for reporting observational studies. To enable optimally prudent prescribing this article presents for primary care physicians and physicians in remote or rural areas without access to such services a comprehensive integration of the data on PIM and PPO medications with the data on the P450 cytochrome isoforms that metabolize these medications. Additionally presented are the medications metabolized by multiple isoforms and medications that inhibit or induce individual or multiple isoforms. The most extensive metabolic activities involve the central nervous system, anxiolytic, antidepressive, antipsychotic, musculoskeletal, and cardiovascular drugs. The P450 cytochrome isoforms that metabolize the most medications are 3A457, 2C9, 2D6, and 2C19 and nearly all central nervous systems medications compete to be metabolized by 3A457. Medications with the largest inducer or inhibitor activity are highlighted and also a list of commonly prescribed medications that are neither PIMs nor PPOs but compete for metabolism by the same isoforms.

## 1. Introduction

The 2017 Global Burden of Disease Study 2017 found that the number of people ≥65 years increased globally by 105% from 327.6 million in 1990 to 673.7 million in 2017 with the highest proportional increases in high-income countries from 121.5 million (12.1% of the population) to 208.6 million (17.5% of the population). The global number of deaths increased from 19.1 million to 32.2 million. The top 10 causes of death for both sexes were ischemic heart disease, stroke, Alzheimer and other dementias, chronic obstructive pulmonary disease, diabetes mellitus, tracheal, bronchial, and lung cancer, and chronic kidney disease and for females hypertensive heart disease, breast cancer, and colon and rectal cancer and for males tuberculosis, cirrhosis and other chronic liver diseases, and stomach cancer [1]. 

The determinants of health worldwide are the country, family, genetic, and social structure into which one is born, lifestyle choices, education, socioeconomic status, inherited and acquired illnesses (particularly multiple chronic comorbidities), and the quality and adequate provision of social services and medical care [2,3]. 

All of the diseases listed above involve extensive medication regimens. The demands for health care and especially for seniors are increasing, with corresponding increases in budgets and numbers of medications. A key question is whether prescribing for seniors is optimally evidence-based. There are two key sources for this evidence base. The first is the American Geriatric Society criteria [4] and the Screening Tool of Older People’s Prescriptions (STOPP)/Screening Tool to Alert to Right Treatment (START) [5] criteria, which are based on systematic literature reviews evaluated by geriatric experts. The second evidence source is the increasing pharmacogenomic database which evaluates how the genetic structure of individuals and the ancestry of individuals and groups affects the metabolism of medications in those individuals and groups. This article combines these evidence sources 

Polypharmacy, defined for those ≥65 as five or more medications [6] is associated with an odds ratio of death of 1.24 (95%CI 1.10, 1.39) for 1–4 medications, 1.31 (1.17, 1.47) for five medications, 1.59 (1.36, 1.87) for 6–9, and 1.96 (1.42, 2.71) for 10 or more medications [7] and also associated with increased numbers of adverse drug events and falls. Judicious deprescribing of polypharmacy is beneficial.

Primary care physicians prescribe medications for most patients and renew medications specialists prescribe. However, guidelines and electronic medical records (EMRs) for community primary care physicians do not provide pharmacogenomic prescribing advice. If prescribers select medications and dosages, wait for patients to report problems, and do not check as appropriate the effects of prescriptions with blood levels, potentially inappropriate medications may be prescribed and patients experience serious drug events (SDEs). 

### 1.1. Purposes of This Article

The purposes are to identify: (1) those medications which the American Geriatrics Society (AGS) and the STOPP criteria assess as “potentially inappropriate medications” (PIMs) and which could be cautiously deprescribed with benefit, and the START criteria as “potential prescribing omissions” (PPOs) which could be new beneficial prescriptions; (2) the pharmacogenomics of the AGS and STOPP PIMs and START PPOs and assess which of these medications are likely to cause SDEs and thus are to be avoided and especially chosen for deprescribing. (3) Encourage physicians as appropriate to order genomic studies and drug levels to guide prescribing and use expert pharmacogenomic advice through their electronic medical record (EMR), EMR decision support tools (DSTs), and specialist pharmacists. (4) Create a table of the PIMs and PPOs which are metabolized by each isoform in the P450 cytochrome system, which will also assist physicians practicing in countries or remoter areas without pharmacogenomic support.

A study investigated how many DSTs with pharmacogenetic data are available in Canada in 2020 and 13 pharmacogenetic tests for psychiatric patients were available at a median cost of CA$499 [8] and a study of four of these DSTs with 1737 patients found that the OR of achieving symptom remission was 1.71 (95%CI 1.17, 2.48; *p* = 0.005) using pharmacogenomic data compared to usual therapy [9]. 

### 1.2. How Frequent Are Potentially Inappropriate Medications (PIMs) and Potential Prescribing Omissions (PPOs)?

PIMs and PPOs prescribed to those 65 and older are most frequently assessed with the American Geriatrics Society (AGS) [4] and the STOPP/START criteria [5]. The 2019 AGS criteria are the expert consensus opinions of a panel of physicians, pharmacists, and nurses based on literature searches in English which focused on “adverse drug events” and “adverse drug reactions.” The 2014 Screening Tool of Older People’s Prescriptions (STOPP) and Screening Tool to Alert to Right Treatment (START) are the expert consensus of 19 geriatricians and pharmacists from 13 European countries and based on literature searches on polypharmacy, inappropriate prescribing, and adverse drug reactions. The update will be available in the late 2020s. 

A systematic review of 62 studies in 32 countries (*n* = 1,854,698) found that patients were exposed to polypharmacy because patients in the community took on average eight medications and hospitalized patients took on average 11 medications and 42–61% of patients had one or more PIMs [10]. 

The potential complications due to PIMs and PPOs were shown in a study of 82,935 patients ≥ 65 who were admitted to the four acute care Calgary hospitals in 2013–18, which found they had a median of three AGS and three STOPP PIMs. An increased risk of readmission within six months of discharge correlated 9% with the number of medications, 15% with the numbers of PIMs, and 6% with the numbers of PPOs. The risk of death within six months correlated 2% with the number of medications, 8% with the numbers of STOPP PIMs, 11% with the numbers of AGS PIMs, and 31% with the numbers of PPOs. Algorithm Rule Mining identified several PIM combinations with a higher likelihood of mortality: the combination of STOPP duplicate drugs from the same class, neuroleptics, and strong opioids had an 8.77 higher risk of death compared to a random combination of STOPP medications, and a 2.36 higher likelihood of readmission for this same set of medications [11]. 

### 1.3. How Frequent Are Serious Drug Events (SDEs) in Seniors?

A key concern is that medications will cause serious drug events and a major purpose of pharmacogenetic-guided therapy is to avoid SDEs. A systematic review of 42 studies of hospitalizations of ≥65 s to August 2015 used the term Adverse Drug Reactions (ADRs) and estimated the average percentage of admissions due to ADRs as 8.7% (95%CI 7.6 to 9.8%; I^2^ = 100%). The most frequent medications involved were nonsteroidal anti-inflammatory drugs (NSAIDs), B-Blockers, and anticoagulants. The assessed studies used a wide variety of causality criteria (14 used the Naranjo causality criteria, seven World Health Organisation-Uppsala Monitoring Centre (WHO-UMC), six Hallas, and seven were not reported) and also a wide variety of ADR definitions (31 used the WHO and six the authors’ own definitions). The quality of studies as assessed by the STROBE criteria ranged from 56% to 96% [12]. 

The largest SDE database is the U.S. Food and Drug Administration Adverse Event Reporting System but its large size and complexity make data mining difficult [13,14], and the latest curated analysis only summarizes reports from 2006 to 2014 [15]. Systematic reviews of SDEs covering multiple countries are rare [16] and most SDE databases are not curated to be useful during the workflow of primary care physicians.

A study of US emergency department visits during 2013–2014 used the term ADRs and assessed that they comprised 4/1000 visits (of whom 27% were hospitalized), and 35% of visits were of ≥65 s (of whom 46% were hospitalized). Anticoagulants, antibiotics, and diabetes medications were involved in 47% of the SDE visits, and included clinically significant SDEs with hemorrhages due to anticoagulants, moderate to severe allergic reactions due to antibiotics, and hypoglycemia with moderate to severe neurological effects due to diabetic medications [17]. 

A study in the Netherlands in 2016 of 856,002 new prescriptions for 45 drugs found that for 24% there was an actionable gene–drug interaction that could lead to an SDE according to the Dutch Pharmacogenetics Working Group (DPWG) guidelines, and in 5.4% of these, there should be a change in dosage or to another medication [18].

### 1.4. Which Are the 10 Most Frequent Medication Classes Prescribed to Seniors in Canada?

Canada has a comprehensive dataset on seniors’ health. The Canadian Institute for Health (CIHI) national dataset of the 10 most frequent medications prescribed to patients ≥65 includes five cardiovascular medications: HMG-CoA reductase inhibitors (statins), angiotensin-converting enzyme (ACE) inhibitors, beta-blocking agents, dihydropyridine calcium channel blockers, and angiotensin II antagonists [19]. Additionally important are opium alkaloids for pain and benzodiazepines for anxiety. Prescribing rates for these medications are similar across all age deciles from 65 to 85+ (Table 1).

### 1.5. Of the Top 10 Medications Prescribed to Seniors in Canada Which Are American Geriatric Society PIMs and for How Many Are the P450 Isoforms That Metabolize Them Known? 

In the CIHI national dataset of the top 10 AGS PIMs in Canada [19], five are for mental health and three for gastroesophageal reflux (Table 2). The P450 isoforms that metabolize them are known only for five of the 10 medications: pantoprazole (2C19, 3A457, and both isoforms are also weak inhibitors); amitriptyline (1A2, 2C9, 2D6, 2C19, 3A457); omeprazole (2C9; 2C19, 3A457; and omeprazole is also an inducer of one and an inhibitor of two isoforms); estrogen (2B6, 2D6, 3A457); and quetiapine (3A457). The P450 isoforms are not known for the remaining five: lorazepam, nitrofurantoin, rabeprazole, zopiclone, and oxazepam (Table 2). 

### 1.6. Which Are the Top 10 Most Frequent Serious Drug Events (SDEs) Associated with Hospitalizations of Seniors in Canada?

The CIHI also lists the 10 medications most commonly associated with hospitalization due to SDEs [19]. Five are cardiac (anticoagulants, beta-adrenoreceptor antagonists, nonthiazide diuretics, thiazide diuretics, and digoxin), two for pain (opioids, NSAIDs), antineoplastics, antipsychotics, and asthma and Chronic Obstructive Pulmonary Disease (COPD) medications. Nine of the medications on this list are AGS and STOPP PIMs and antineoplastics would be appropriately classed as AGS and STOPP PIM drugs (Table 3). 

For six of these medications associated with SDEs, the P450 isoforms are known: for anticoagulants, five isoforms metabolize clopidogrel and ticlopidine (1A2, 2B6, 2C9, 2C19, 3A457, and both anticoagulants are also inhibitors for three isoforms), including four metabolizing warfarin (1A2, 2C9, 2C19, and 3A457), three P450 isoforms metabolizing aspirin, and dipyridamole (2B6, 2C8, 2E1). Warfarin, in addition, is metabolized by four VKOR1 genes and it is strongly advised to use a DST to manage initial warfarin dosing and if new medications are prescribed to the patient. Opioids are metabolized by P450 isoforms 2B6, 2D6, and 3A457; of the NSAIDs, ibuprofen is metabolized by six isoforms, indomethacin by five, and the remainder including aspirin by three (2B6, 2C8, and 2E1). B-blockers are metabolized by 2C9, 2C19, and 2D6, and diuretics by 1A2, 2D6, and 3A457. Thus, these medications, which are among the top 10 associated with SDEs, are metabolized by multiple P450 isoforms, and many other medications will compete to be metabolized by these isoforms. Knowledge of these pharmacogenomic data is important when prescribing these medications. The combination of being PIMs, on the SDE list, and having complex pharmacodynamics makes them good candidates for prudent deprescribing. 

The US Veterans Affairs publishes a large database of SDEs. The predominance among their top 10 SDEs of cardiovascular medications is also to be noted: angiotensin II receptor blockers (ARBs), statins, warfarin, amlodipine, and hydrochlorothiazide with similar rankings across all ≥65 age deciles [20]. 

## 2. Metabolism of Medications by the P450 Enzyme System

Medications can have a long period of activity in the body for multiple reasons. Key causes are if they are either lipophilic medications which are strongly bound to plasma proteins and not filtered at the glomerulus, or if they are unionized or partly ionized at physiological pH and thus easily reabsorbed from nephrons. The supergene Cytochrome P450 CYP family has 57 genes, and 12 of these genes code for Stage 1 reactions in which 75% of prescription medications are transformed into a more polar and easily excreted form by adding functional groups such as -OH, -NH_2_, or -SH. Many Phase 1 products are still not excreted rapidly and so are converted in P450 Phase 2 reactions to more highly polar forms by adding sulfuric acid, acetic acid, glucuronic acid, or an amino acid. The term cytochrome is used because the microsomal hemoprotein enzyme during the oxidation–reduction reaction in its reduced ferrous form binds to carbon monoxide and absorbs light maximally at 450 nm [21]. 

Humans have only recently used medications and, therefore, there has been no evolutionary pressure to deselect Phase 1 or 2 reactions which are now highly polymorphic. Many have proven to have adverse consequences for medication metabolism [22] because of their wide range of metabolic rates. Individuals can have two nonfunctioning P450 CYP alleles with no enzymatic activity (patients are designated “poor metabolizers”), a nonfunctioning plus a normal allele with 50% enzymatic activity (“intermediate metabolizers”), two normal alleles with 100% enzymatic activity (“normal metabolizers”), one or two “gain of function” alleles with 120–150% more enzymatic activity, or duplication of genes (“ultrarapid metabolizers”) [23]. 

In addition to having varying metabolic rates, there are three complications of this variety of alleles between individuals and ancestries. Firstly, medications may compete as substrates to be metabolized by the same CYP450 alleles and thus delay metabolism. Secondly, medications can be inducers of one or more CYP450 alleles and cause decreased blood levels and undertreatment by other medications using that isoform. Thirdly, medications can be inhibitors of one or more isoforms and cause increased blood levels, overtreatment, and possible SDEs. The CYP2D6 gene is the most polymorphic P450 isoform and has more than 100 alleles [23].

There are thus multiple factors that complicate optimal prescribing. Inappropriate prescribing of PIMs and PPOs may cause SDEs, rehospitalizations, or death. There is a large genetic variability in the metabolism of PIMs, PPOs, and other medications with additional variations due to the effects of sex, age, ancestry, and comorbidities. 

The solution to this problem is three-fold: avoid prescribing PIMs and PPOs wherever feasible, prudently deprescribe PIMs and PPOs especially those with complex adverse metabolism by the P450 cytochrome system, and use EMRs with pharmacogenomic CDTs to optimize prescribing. However, current pharmacogenomic guidelines are mostly used by specialists and focus principally on prescribing for mental health, cardiovascular disease, GI, infectious diseases, musculoskeletal diseases, pain, malignancy, and HIV. 

Because primary care physicians prescribe about 70% of all patients’ medications there is a need to provide them with pharmacogenetic information and also to physicians in remote and rural areas and in countries without access to pharmacogenomic testing and EMR Decision Support Tools (DSTs). This article focuses on prescribing for patients ≥65 and to avoid prescribing problems provides tables that can either be copied into an EMR or computer tablet or consulted on paper by physicians in remote or rural areas. 

## 3. Materials and Methods 

### 3.1. Study Design

**Figure 1:**
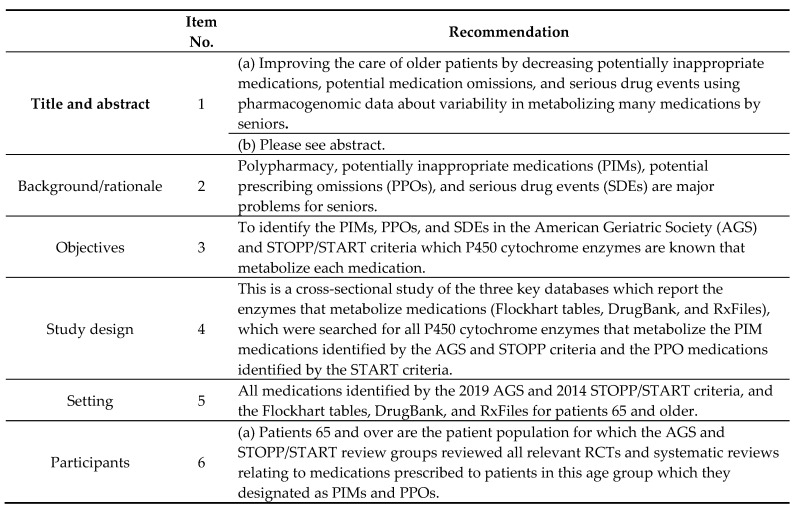

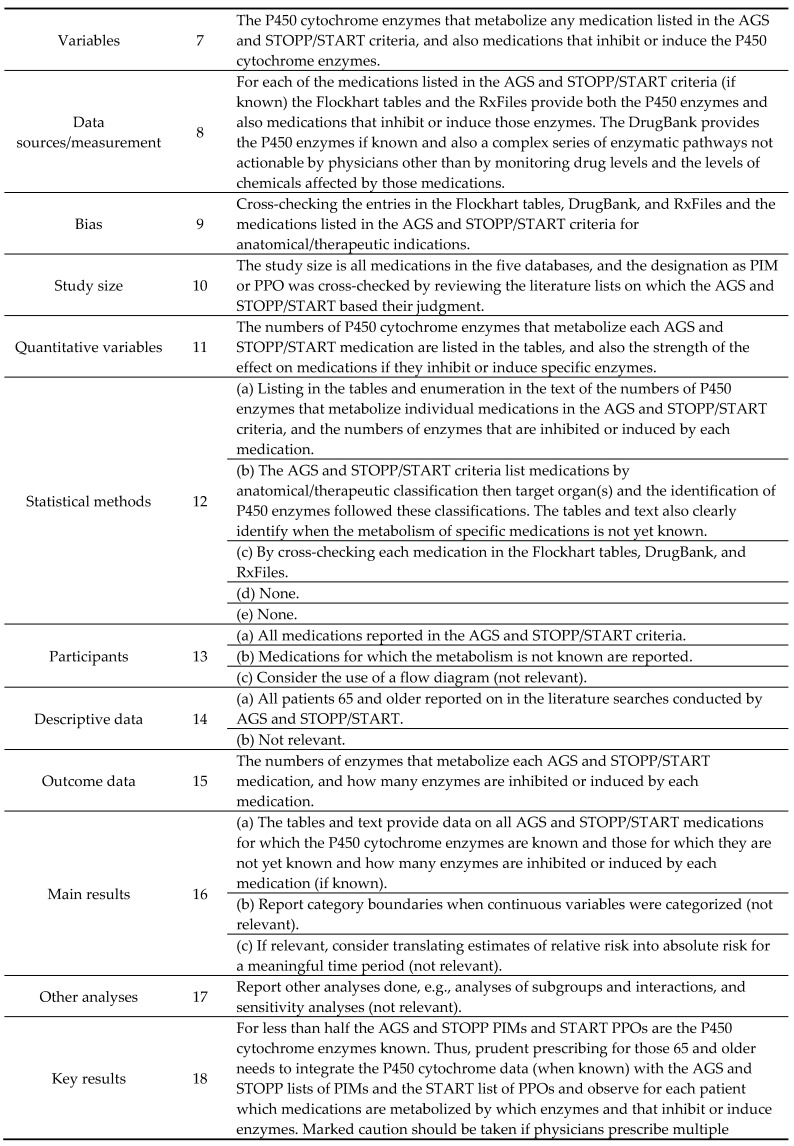

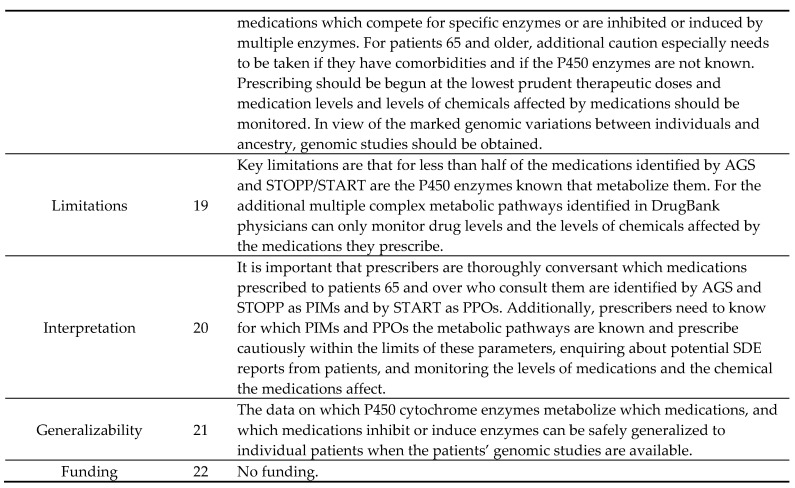
Strengthening the Reporting of Observational Studies in Epidemiology (STROBE) statement—Checklist of items that should be included in the reports of cross-sectional studies.

The study design is a cross-sectional analysis of AGS and STOPP PIM and START PPO databases integrated with three P450 cytochrome enzyme databases (Flockhart tables, DrugBank, and Rx Files). The data are reported (Figure 1) using the Strengthening the Reporting of Observational Studies in Epidemiology (STROBE) Statement: guidelines for reporting observational studies [24].

### 3.2. The Key Sources of Pharmacogenomic Data

Pharmacogenomic data are provided on complex websites and consulting them would not be feasible during busy office practice for primary care physicians. Only physicians working in very large health care systems currently have access to free comprehensive pharmacogenomic EMRs, EMRs with pharmacogenomic DSTs, and expert pharmacist advice. Therefore, this article abstracts from these complex data sources the key information primary care physicians can then save and search as Word or paper documents. The tables will enable ready identification of: (a) which medications compete to be metabolized by the same isoform; (b) which medications induce specific isoforms to metabolize faster so that blood levels of other medications are subtherapeutic; and (c) which medications inhibit isoforms to metabolize more slowly so that blood levels of other medications become too high and potentially toxic. 

The key sources of pharmacogenomic data are DrugBank [25] and the Flockhart tables [26] from the Department of Medicine at Indiana University (which include the medications most likely to be prescribed by primary care physicians). 

The Flockhart tables report pharmacogenomic data for nine key P450 cytochromes that metabolize medications. They are particularly helpful because they also report which medications are inhibitors of specific P450 cytochrome isoforms (thus slowing drug metabolism and resulting in higher drug levels, overtreatment, and potentially toxic levels from prodrugs) or inducers (thus speeding up metabolism and resulting in lower drug levels) [26]. DrugBank tables are a comprehensive medication chemical database and report multiple complex pathways including the P450 cytochromes for each medication [25]. DrugBank is based on clinical trial reports and journal publications but also on drug manufacturer labels and there may be minor differences from the Flockhart tables. Both databases are freely available on the Internet and maintained by volunteers. RxFiles [27] provides a convenient one-page list for primary care physicians and is based on their literature searches. All three databases are free of influences from pharmaceutical companies. 

## 4. Results

For each of the medications listed in the AGS and STOPP/START criteria (if known) the Flockhart tables and the RxFiles provide both the P450 enzymes and also medications that inhibit or induce those enzymes. The DrugBank provides the P450 enzymes if known and also a complex series of enzymatic pathways not actionable by physicians other than by monitoring drug levels and the levels of chemicals affected by those medications. 

The AGS and STOPP/START criteria list medications by anatomical/therapeutic classification then target organ(s) and the identification of P450 enzymes followed these classifications. The tables and text also clearly identify when the metabolism of specific medications is not yet known.

### 4.1. STOPP PIMs for Which the P450 Cytochrome Isoforms That Metabolize Them Are Known

This analysis (Table 4) focuses on nine CYP cytochrome isoforms that metabolize 75% of all medications. Some medications may be prescribed within multiple anatomic/therapeutic categories. Table 4 follows the anatomic then therapeutic categories used by the STOPP/START criteria. When prescribing the following need to be borne in mind:

1. The busiest isoforms (in order) are (Table 4): 3A457 (97 medications), 2C9 (96 medications), 2D6 (75 medications), 2C19 (48), 1A2 (38), and 2C8 (29), 2B6 (24), and 2E1 (22). If prescribers are providing multiple medications for patients with many chronic comorbidities, thought needs to be given whether they are overloading specific isoforms.
2. Because patients may request multiple central nervous system medications including antidepressants, anxiolytics, sedatives, hypnotics, psychiatric, and skeletal muscle relaxant drugs this group will likely involve the most extensive and complex metabolic activities, with the greatest range of inhibitor and inducer activity and the greatest risk of SDEs. Of the anticholinergics, diphenhydramine is metabolized by six isoforms and inhibits one. Of the antidepressants, the tricyclics are metabolized by five isoforms; the SSRIs and SNRIs by four, escitalopram and fluvoxamine are inhibitors of four, fluvoxamine is an inhibitor of three, and paroxetine an inhibitor of two. The antipsychotics are mostly metabolized by two isoforms (2D6 and 3A457) but olanzapine by five. Of the barbiturates, butalbital is metabolized by five isoforms and phenobarbitone is an inducer of three isoforms. Of the benzodiazepines, diazepam is metabolized by four isoforms. The antiepileptic carbamazepine is metabolized by five and induces four isoforms, phenytoin is metabolized by five and induces two, and phenobarbitone by one isoform but induces three. Of the smooth muscle relaxants, orphenadrine is metabolized by five isoforms and methocarbamol by four. The 3A457 isoform metabolizes all CNS medications and it would be very easy to overload it. Great caution is thus required in prescribing for patients with multiple CNS complaints.
3. Cardiac and antiplatelet/anticoagulant medications require great caution to avoid causing SDEs because they are metabolized by and inhibit a wide variety of isoforms. Amiodarone is an inhibitor of four isoforms. Ticlopidine is metabolized by five isoforms and inhibits four. Clopidogrel is metabolized by five and inhibits two. Warfarin is metabolized by four and also by the genes VKORC1 and CYP4F2, aspirin by three, and apixaban, rivaroxaban, and ticagrelor by 3A457.
4. The proton-pump inhibitors are also metabolized by multiple isoforms. Omeprazole is metabolized by three isoforms (2C9, 2C19, and 3A457, and also induces 1A2 and inhibits 2C19 and 3A457) and esomeprazole is similar.
5. Although theophylline is rarely used as a respiratory medication but may be prescribed in some countries and it is metabolized by five isoforms
6. The musculoskeletal drugs are also metabolized by multiple isoforms. Ibuprofen is metabolized by six isoforms, indomethacin by five, and naproxen by four. The remainder are metabolized by three isoforms (2B6, 2C8, and 2E1). Of the Cox-2 inhibitors, celecoxib is metabolized by six isoforms and meloxicam by four.
7. The narcotics all are metabolized by 2D6 and 3A457 and meperidine and tramadol also by 2B6. When patients seek multiple narcotics, this is particularly important to remember as a reason for declining.

For several STOPP/START anatomical and therapeutic categories it is not known if P450 isoforms metabolize these:

1. For the cardiovascular system not known for any of the 12 ACE inhibitors, 5 of 11 beta-blockers, two of six calcium channel blockers, all four alpha-blockers, two of the three anticholinesterase inhibitors, and for digoxin.
2. For the central nervous system, none of the anti-Parkinson medications.
3. For the GU and GI systems, 13 antimuscarinics and none of the antispasmodics.
4. For the respiratory system, the antimuscarinic bronchodilators.
5. For the musculoskeletal system, none of the five bisphosphonates.

Many medications are metabolized by multiple complex enzyme pathways other than the P450 isoforms. DrugBank [25] often provides up to six such complex metabolic pathways, each pathway consisting of 6 to 50 enzymes. Some of these pathways include P450 isoforms but it is unclear what percentage of the metabolic activity they provide. The diuretics are an example because they are metabolized by complex pathways beginning with solute carrier families 12 and 22, the sodium/potassium transporting ATPase subunit alpha-1, and multiple other enzymes that are not currently actionable by physicians other than by measuring blood levels of the medications and the chemicals such as potassium or sodium they are intended to regulate. 

The AGS PIMs and the STOPP/START PIMs and PPOs include both specific individual medications and classes of medications and to complete the class lists in the AGS and STOPP/START publications [4,5] physicians need to add individual medications relevant to the country they work in. 

### 4.2. American Geriatric Society PIMs for Which the P450 Cytochrome Isoforms That Metabolize Them Are Known

The AGS PIM criteria contain many of the same medications as STOPP but are organized into an initial comprehensive anatomical/therapeutic table of PIMS to be avoided and then very useful tables of medications to be used with caution, medications that increase drug–disease interactions, medications to be avoided, or dosage reductions according to kidney function, and a final very helpful table of 56 medications with strong anticholinergic properties to be avoided [4]. AGS PIMs are listed in Table 5 below and the anatomical/therapeutic categories for which the P450 isoform is known are listed. It is notable that for a substantial number of anatomic/therapeutic categories the P450 isoforms boxes are empty indicating that it is not yet known if they are metabolized by P450 enzymes. 

Particularly helpful is the AGS advice about: (1) which medications to avoid, (2) medications which increase drug–disease interactions, and (3) Medications with strong anticholinergic properties [4].

1. Medications to be avoided [4] (AGS Table 2). Within this group the P450 isoforms that metabolize some of the following medications are known, and can be used as additional information leading to further caution:
  - Five first-generation antihistamines (the P450 isoforms of six of these are known);
  - Ten highly anticholinergic antidepressants (the P450 isoforms of six are known);
  - Seven barbiturates (the P450 isoforms of five are known);
  - Thirteen benzodiazepines (the P450 isoforms of two are known);
  - Androgens and estrogens (P450 isoforms are known);
  - Three long-acting sulfonylureas (P450 isoforms of two are known);
  - Proton-pump inhibitors (P450 isoforms of four are known);
  - Eighteen NSAIDs (P450 isoforms of 12 are known);
  - Six skeletal muscle relaxants (P450 isoforms of five are known).
  P450 isoforms are not known for:
  j. Two anti-Parkinsonian agents (benztropine and trihexyphenidyl);
  k. Three peripheral alpha-agonists;
  l. Four CNS alpha-agonists;
  m. Cardiac medications: disopyramide, dronedarone, digoxin, and amiodarone;
  n. Three nonbenzodiazepine, benzodiazepine receptor agonist hypnotics.
2. Medications that increase drug–disease interactions. AGS advises avoiding these [4] (Table 3). Medications and the P450 isoforms that metabolize them are known for some and listed in Table 5 below.
  - For patients with heart failure:
    1. Eighteen NSAIDs (P450 isoforms of 12 are known);
    2. The two Cox-2 inhibitors celecoxib and meloxicam (P450 isoforms are known);
    3. amiodarone, rosiglitazone, pioglitazone, and troglitazone (P450 are known but for cilostazol and dronedarone are not known).
  - For patients with syncope:
    1. Three acetylcholinesterase inhibitors (P450 is known for donepezil);
    2. Seven peripheral alpha-1 blockers (P450 are not known);
    3. Six tricyclics (P450 are known);
    4. Chlorpromazine (P450 is not known).
  - For patients with central nervous system problems of delirium and dementia:
    1. 56 medications with strong anticholinergic properties (P450 isoforms are known for 22);
    2. Fourteen antipsychotics (P450 are known for 11);
    3. Four benzodiazepines (P450 are known for two);
    4. Four nonbenzodiazepine benzodiazepine receptor hypnotics (P450 is known for one).
  - For patients with falls in addition:
    1. Four H2-receptor antagonists (P450 are known for cimetidine and ranitidine);
    2. Anticonvulsants (P450 are known for five).
  - For patients with Parkinson’s:
    1. Fourteen antipsychotics (P450 are known for 11);
    2. Antiemetics (P450 are known for two);
    3. Medications with strong anticholinergic properties. AGS ([4], AGS Table 7) recommends avoiding these and provides a comprehensive list of 56 medications with strong anticholinergic properties used in multiple anatomic/therapeutic classes: antiarrhythmics, antidepressants, antiemetics, antihistamines, antimuscarinics, antiparkinsonian agents, antipsychotics, antispasmodics, and skeletal muscle relaxants [4].

The P450 metabolic enzymes are known for only some of the STOPP PIMs and START PPOs and empty cells in Table 5 indicate those for which it is not yet known if they are metabolized by P450 enzymes. 

### 4.3. For How Many START PPOs Are the P450 Isoforms Known? 

The dominant role of isoform 3A457 is notable. The START PPO list focuses especially on cardiovascular, central nervous system, and musculoskeletal medications (Table 6). Cardiovascular medications use a wide variety of P450 isoforms. For a substantial number of anatomic/therapeutic categories if there is no listing for a P450 isoform then it is not yet known. The importance of identifying PPOs and prescribing them is shown by a study of 82,935 patients ≥65 admitted to the four acute care Calgary hospitals in 2013–2018 which found a 31% correlation of PPOs with death within six months of discharge, indicating unmet needs [11].

### 4.4. Genetic Influences on Cell Influx and Efflux Pumps

Once medications have been passed into the bloodstream by the P450 and other metabolic enzymes they are enabled to enter and leave cells by influx and efflux pumps [27] which are encoded by different genes. The organic anion-transporting polypeptides (OATPs) are influx pumps and enable e.g., antibiotics (ciprofloxacin and erythromycin) and ARBs to enter cells. The OATPs are inhibited by the antibiotics clarithromycin, erythromycin, ketoconazole, and rifampin. The *p*-glycoprotein efflux pump removes important cardiovascular medications (amiodarone, apixaban, dabigatran, digoxin, diltiazem, edoxaban, rivaroxaban, and verapamil) and antibiotics (erythromycin, posaconazole, and rifampin) are induced by carbamazepine, phenobarbital, dexamethasone, phenytoin, primidone, rifampin, and St John’s Wort, many of which are inhibitors or inducers of multiple P450 isoforms. Macrolides and antifungals can be important in causing ADRs because of their transport by influx and efflux pumps (Table S1). 

### 4.5. Frequently Prescribed Medications Which Are Neither PIMs nor PPOs

It is important to identify which medications are frequently prescribed but are neither PIMs nor PPOs but also compete for metabolism by P450 isoforms because this affects the ultimate work burden of each isoform and the drug levels of medications metabolized by them (Table S2). Several of these medications are important lifestyle choices: caffeine is metabolized by five isoforms, cocaine is metabolized by two isoforms and inhibits a third, and ethanol is metabolized by 2E1 and also induces it (Table S3).

Several medications in these lists are metabolized by multiple isoforms: methadone by seven, cimetidine by four and is an inhibitor of four; fluconazole by four and inhibits two; oxcarbazepine, tamoxifen, and terbinafine by four; voriconazole by three and inhibits four; and rifampin is an inducer of six isoforms and isoniazid of three. The preponderance of antifungals as strong inhibitors is of note. 

## 5. Discussion 

The American Geriatric Society and the STOPP/START groups of geriatricians and pharmacists assessed comprehensive lists of PIMs and PPOs from randomized controlled trials and systematic reviews of how patients ≥65 respond to individual medications and experience SDEs attributable to individual medications and combinations of medications [4,5]. Detailed prescribing advice is also provided about avoiding drug–drug interactions, exacerbations of diseases by drug–disease interactions, avoiding further impairment of already decreasing renal function, and avoiding medications involved in falls. 

DrugBank [25], the Flockhart tables [26], and RxFiles [27] report the biological and chemical studies report which P450 isoforms metabolize the AGS and STOPP PIMs and the START PPOs if known, but many remain unknown. The enzymes which control the important pumps which pump medications and chemicals into and out of cells are also partially known. 

The DrugBank studies record both the metabolism of many medications by multiple P450 cytochrome isoforms and also multiple complex pathways for some medications each including from 6 to 60 enzyme pathways which do not include P450 cytochrome isoforms [25] and the only actionable steps for physicians are to obtain blood levels of the medications and any chemicals (e.g., potassium or sodium) the medications are intended to regulate. 

This article integrates the data about PIMs, PPOs, and SDEs with the DrugBank [25], Flockhart Tables [26], and RxFiles [27] pharmacogenomic data to provide an overall prescribing plan, emphasizing high-risk situations where there are multiple medications, inducers, and inhibitors affecting multiple P450 cytochromes, and also medications for which the P450 cytochromes are as yet unknown. Because of the declining ability of seniors to metabolize medications additional caution is needed in prescribing for these individuals who are subject to impaired organ function, confusion, and falls. 

## 6. Limitations

The P450 cytochrome isoforms that metabolize many frequently prescribed medications (Table 3, Table 4, Table 5 and Table 6 and Tables S1–S3) are known from decades of basic biochemistry research reported in the DrugBank [25] and Flockhart tables [26] but many enzyme pathways are unknown. For the many medications for which multiple enzymatic pathways are recorded in DrugBank and are not P450 pathways, the only options for physicians are to measure the end results of these complex pathways by assessing blood concentrations either of the medications or of the effects of medications (e.g., electrolytes such as sodium and potassium).

Thus, prudent prescribing for those 65 and older needs to integrate the P450 cytochrome data (when known) with the AGS and STOPP lists of PIMs and the START list of PPOs and observe for each patient which medications are metabolized by which enzymes and that inhibit or induce enzymes. Marked caution should be taken if physicians prescribe multiple medications which compete for specific enzymes or are inhibited or induced by multiple enzymes. For patients 65 and older, additional caution especially needs to be taken if they have comorbidities and if the P450 enzymes are not known. Prescribing should be begun at the lowest prudent therapeutic doses and medication levels and levels of chemicals affected by medications should be monitored. In view of the marked genomic variations between individuals and by ancestry genomic studies should be obtained.

Asking patients and their carers about their ideas and experiences about their medications is essential. There are no studies yet of the feasibility of patient enthusiasm to educate patients about the complexities of the pharmacogenomics of their specific medications. 

There are major educational, organizational, and financial complexities of introducing comprehensive pharmacogenomic prescribing into health care systems. How to accomplish this and integrate patients into the prescribing team is the subject of a future article.

## 7. Conclusions 

PIMs, PPOs, and SDEs are important problems for seniors. A study of 82,935 patients ≥65 admitted to the four acute care Calgary hospitals 2013-18 found they were admitted with a median of four medications, three STOPP PIMs and two AGS PIMs, 23% had five or more AGS PIMs, and 31% five or more STOPP PIMs. They were discharged with a median of nine medications [11]. The correlation of PPOs with a 31% increased risk of mortality within six months of hospital discharge indicates the importance of being prescribed these missing medications and also of educating their physicians to do so.

Nine P450 cytochrome isoforms metabolize 75% of medications. Medications are metabolized by several isoforms, some by up to seven isoforms, and many medications are also inducers of isoforms (resulting in lower levels of medications metabolized by those isoforms and thus undertreatment) or are inhibitors of isoforms (resulting in higher levels of medications metabolized by those isoforms, overtreatment and toxicity with prodrugs).

It is thus essential to integrate data about PIMs and PPOs with biological and chemical data on which P450 isoforms metabolism data and which are strong or weak inhibitors or inducers. It is also important to identify which medications are not metabolized by P450 isoforms but by complex enzyme sequences including such as solute carriers and sodium/potassium transporting ATPase subunit alpha-1 (part of the thiazide metabolism pathway) which require monitoring of blood levels of e.g., electrolytes.

It is also important to understand how medications are transported into cells by organic anion-transporting polypeptides and exported by *p*-glycoprotein efflux pumps which are also influenced by patient genetics, and which medications are inducers and which are inhibitors of these pumps (Table S1). 

There are also many medications which are not designated as PIMs or PPOs but which are metabolized by, induce, or inhibit the same isoforms as the PIMs and PPOs. It is important not to overload these isoforms so that combinations of medications, inducers, or inhibitors result in undertreatment, overtreatment, or severe drug events (Table S2).

Physicians need to ensure that: (1) they know which medications are PIMs or PPOs and (2) they have the pharmacogenomic data for each PIM or PPO so that they can avoid prescribing likely to result in inadequate therapy and ADEs. (3) They avoid sending multiple medications though the same P450 isoform so that the isoform is overloaded and inadequate therapeutic levels are achieved, and (4) That they know which medications induce or inhibit specific isoforms so that they can avoid inducing or inhibiting isoforms that then result in dangerous drug levels for multiple medications using those isoforms. 

Medications are often prescribed at the end of a visit after multiple other problems have been addressed. The patient may conclude by asking “I’d like all my medications renewed” yet the physician has run out of time. A key concept of this article is that prescribing is an activity of primary care physicians that can confer the greatest benefits in prevention and treatment but also involves substantial risks. Time should be set aside for a special visit periodically to discuss medications. Having Table 4, Table 5 and Table 6 and Tables S1–S3 available during prescribing either on the EMR or on paper will enable primary care prescribers to give the patient an overview of how to improve prescribing for seniors and the SDEs to be avoided. The patients need to be able to identify PIMs and PPOs which may confer risks for them. The PIMs and PPOs which are competing to be metabolized by individual or multiple groups of P450 cytochromes need to be discussed to optimize the preferred medication list (Table 4, Table 5 and Table 6).

Prudent deprescribing could take place over several visits if necessary. Thoughtful deprescribing strategies are provided in Geri-Rx Files [28]. 

The physician can show the detailed advice tables in the 2019 AGS criteria [4] as an authoritative source to discuss alternatives and a deprescribing plan. 

Because humans have used medications only for a few decades, the P450 cytochrome isoforms have not been subject to evolutionary selection by medications and have developed a huge range of genetic variability especially by ancestry. This topic and how to use DSTs to incorporate data about genetic variability and variability due to ancestry in prescribing is the subject of a subsequent article. 

## Figures and Tables

**Table 1:** Top 10 drug classes prescribed to seniors, by the rate of use and chronic use, Canada, 2016 [19].

Drug Class	Common Uses		Rate of Use, Chronic Use	By Age Group		
		Rate of Use	Rate of Chronic Use (Defined by 2016 AGS Criteria)	65 to 74	75 to 84	85+
HMG-CoA reductase inhibitors (statins)	High cholesterol	48.4%	43.5%	47.5%	53.1%	41.8%
Proton-pump inhibitors (PPIs)	Gastroesophageal reflux disease, peptic ulcer disease	32.1%	23.5%	28.3%	35.5%	39.0%
Angiotensin-converting enzyme (ACE) inhibitors, excluding combinations	High blood pressure, heart failure	24.5%	21.1%	22.5%	26.7%	27.2%
Beta-blocking agents,	selective High blood pressure, heart failure, angina (chest pain)	23.5%	20.6%	18.7%	27.9%	32.5%
Dihydropyridine calcium channel blockers	High blood pressure	21.9%	18.8%	17.8%	25.5%	29.8%
Thyroid hormones	Hypothyroidism	19.1%	17.9%	16.0%	20.8%	26.6%
Angiotensin II antagonists, excluding combinations	High blood pressure, heart failure	15.7%	13.8%	13.8%	18.1%	17.8%
Natural opium alkaloids	Management of moderate to severe pain	15.1%	2.5%	14.2%	15.4%	17.9%
Biguanides	Diabetes	14.9%	12.9%	15.3%	16.0%	10.9%
Benzodiazepine derivatives	Agitation, anxiety, insomnia, seizures	12.9%	6.1%	11.1%	14.4%	16.9%

**Table 2:** Top 10 chemicals from American Geriatric Society Beers list prescribed to seniors, by the rate of use and chronic use, Canada [19].

Chemical	Indicated Uses	AGS Beers Criteria Rationale (Potential Harm)	Rate of Use	Rate of Chronic Use (Defined by 2016 AGS Criteria)
Pantoprazole (PPI) (>8 weeks)	Gastroesophageal reflux disease, peptic ulcer disease	Clostridium difficile infection, bone loss, fractures	13.2%	10.3%
Lorazepam	Anxiety, insomnia	Cognitive impairment, delirium, falls, fractures	8.8%	3.6%
Nitrofurantoin	Antibiotic to treat urinary tract infection	Pulmonary toxicity, hepatotoxicity, peripheral neuropathy	5.0%	0.1%
Rabeprazole (PPI) (>8 weeks)	Gastroesophageal reflux disease, peptic ulcer disease	Clostridium difficile infection, bone loss, fractures	4.3%	3.5%
Amitriptyline	Depression	Sedation, orthostatic hypotension	2.9%	1.8%
Quetiapine	Schizophrenia, bipolar disorder	Cognitive decline, stroke, mortality	2.8%	1.7%
Omeprazole (PPI) (>8 weeks)	Gastroesophageal reflux disease, peptic ulcer disease	Clostridium difficile infection, bone loss, fractures	2.7%	2.2%
Zopiclone	Insomnia	Cognitive impairment, delirium, falls, fractures	2.4%	1.5%
Oxazepam	Anxiety, insomnia	Cognitive impairment, delirium, falls, fractures	2.4%	1.4%
Estradiol (oral/topical patch)	Menopause	Potential carcinogen (breast and endometrium)	2.1%	1.2%

**Table 3:** Top 10 drug classes most commonly associated with seniors’ Adverse Drug Reaction (ADR)-related hospitalizations, 2006–2007 to 2010–2011 [19].

Drug Class Most	Common Uses	Most Common Diagnosis Related to Hospitalization	Percentage of ADRs
Anticoagulants	Heart attack and stroke prevention	Hemorrhagic disorders due to circulating anticoagulants	12.6%
Antineoplastic drugs	Cancer	Neutropenia	12.1%
Opioids and related analgesics	Pain management	Constipation	7.4%
Glucocorticoids and synthetic analogs	Asthma	Chronic obstructive pulmonary disease with acute lower respiratory infection	6.9%
NSAIDs (excluding salicylates)	Arthritis, pain management, inflammation	Gastric ulcer, chronic or with hemorrhage	4.9%
Beta-adrenoreceptor antagonists, not elsewhere classified	Heart failure, high blood pressure, angina	Bradycardia	4.6%
Nonthiazide (low-ceiling) diuretics	Heart failure, high blood pressure	Hypo-osmolality and hyponatremia	3.6%
Thiazide diuretics	High blood pressure	Hypo-osmolality, hyponatremia	3.2%
Cardiac-stimulant glycosides (e.g., digoxin)	Heart failure, arrhythmia	Bradycardia	3.1%
Antipsychotics	Symptoms of dementia, schizophrenia, bipolar disorder	Disorientation	2.7%

**Table 4:** Screening Tool of Older People’s Prescriptions (STOPP) 2015 criteria for Potentially Inappropriate Medication Use in older adults for which the P450 cytochrome isoform is known. Sources: **Bold print** = DrugBank [25]; Regular print = Flockhart Table [26]; *Italic Print* = Rx Files. 12th ed. [27] (p 211). If a medication name is given it indicates it is a substrate metabolized by the P450 cytochromes in the columns listed. If a medication is also an inducer of individual P450 cytochromes the word inducer is used. If a medication is an inhibitor of individual P450 cytochromes the word inhibitor is used. (Inhibitors increase levels of medications metabolized by the same isoform. Inducers decrease levels of medications metabolized by the same isoform). However, if they metabolize a prodrug (e.g., codeine) the result is higher levels of its metabolites (e.g., morphine).

STOPP Cardiovascular System								
Medication	P450 Cytochrome Isoforms							
	1A2	2B6	2C8	2C9	2C19	2D6	2E1	3A457
Amiodarone	Inhibitor			Moderate inhibitor		Weak inhibitor		*3A4 Amiodarone*; Inhibitor
Diltiazem								Diltiazem, also moderate inhibitor; *3A4* Diltiazem
Torsemide			Torsemide	Torsemide				
Verapamil	Verapamil; Verapamil							Verapamil, also moderate inhibitor; *3A4 Verapamil*
**Potassium-sparing diuretics**								
Eplerenone								Eplerenone
Triamterene	Triamterene							
**B-blockers**								
Bisoprolol						*Bisoprolol*		
Labetalol					Labetalol			
Metoprolol						Metoprolol; *Metoprolol*		
Propranolol	Propranolol; *Propranolol*				*Propranolol*	*Propranolol*		
Timolol						Timolol; *Timolol*		
**Angiotensin-II receptor blockers**								
Irbesartan				Irbesartan; *Irbesartan*				
Losartan				Losartan; *Losartan*				
Valsartan				*Valsartan*				
**STOPP Antiplatelet/anticoagulant drugs**								
	**1A2**	**2B6**	**2C8**	**2C9**	**2C19**	**2D6**	**2E1**	**3A457**
Apixaban								*3A4 Apixaban*
Aspirin >325 mg/d, also **CYP 2J2, 2U1, 4A11, and 4F8**		**Aspirin**	**Aspirin**				**Aspirin**	
Clopidogrel	*Clopidogrel*	**Clopidogrel** inhibitor		**Clopidogrel;** Clopidogrel, also inhibitor	**Clopidogrel;** Clopidogrel; *Clopidogrel*			**Clopidogrel 3A4,3A5** Clopidogrel
Dipyridamole, also **CYP 2J2, 2U1, 4A11, and 4F8**		**Dipyridamole**	**Dipyridamole**				**Dipyridamole**	
Rivaroxaban								*3A4 Rivaroxaban*
Ticagrelor								*3A4 Ticagrelor*
Ticlopidine	**Ticlopidine;** inhibitor	**Ticlopidine**; inhibitor		**Ticlopidine**	**Ticlopidine**; inhibitor	Inhibitor		**Ticlopidine 3A4, 3A5**
Warfarin	Warfarin; *Warfarin*			s-Warfarin; *Warfarin*	r-Warfarin; *Warfarin*			*3A4 Warfarin*
For non-cyclooxygenase-selective NSAIDs and Cox-inhibitors see musculoskeletal drug list								
**STOPP Central nervous system and psychotropic drugs**								
	**1A2**	**2B6**	**2C8**	**2C9**	**2C19**	**2D6**	**2E1**	**3A457**
**Anticholinergics: First-generation antihistamines**								
Chlorpheniramine						Chlorpheniramine, also inhibitor		Chlorpheniramine
Clemastine						inhibitor		
Diphenhydramine	*Diphenhydramine*		**Diphenhydramine**	**Diphenhydramine**; *Diphenhydramine*	**Diphenhydramine**; *Diphenhydramine*	*Diphenhydramine;* inhibitor		**Diphenhydramine 3A4**
Hydroxyzine						inhibitor		
Promethazine						Promethazine, also inhibitor **Promethazine**		**Promethazine**
**Antidepressants: Tricyclics**								
Amitriptyline	Amitriptyline; *Amitriptyline*			Amitriptyline; *Amitriptyline*	Amitriptyline; *Amitriptyline*	Amitriptyline; *Amitriptyline*		Amitriptyline; *3A4 Amitriptyline*
Clomipramine	Clomipramine; **Clomipramine;** *Clomipramine*				Clomipramine; **Clomipramine;** *Clomipramine*	Clomipramine, also inhibitor **Clomipramine;** *Clomipramine*		**Clomipramine**
Desipramine	**Desipramine;** *Desipramine*				**Desipramine;** *Desipramine*	**Desipramine** Desipramine;		**Desipramine 3A4**
Doxepin >6 mg/d	Doxepin; **Doxepin**			Doxepin; **Doxepin**; *Doxepin*	Doxepin; **Doxepin;** *Doxepin*	Doxepin, also inhibitor; **Doxepin;** *Doxepin*		Doxepin; **Doxepin 3A4**
Imipramine	Imipramine; **Imipramine;** *Imipramine*				Imipramine; **Imipramine**	Imipramine; **Imipramine**		**Imipramine 3A4**
Nortriptyline						Nortriptyline; *Nortriptyline*		
**Antidepressants: SSRIs**								
Citalopram	Weak inhibitor				Citalopram, also weak inhibitor; **Citalopram;** *Citalopram*	Citalopram, also weak inhibitor; **Citalopram**		Citalopram; **Citalopram 3A7;** *3A4 Citalopram*
Escitalopram					Escitalopram; *Escitalopram*	Escitalopram, also weak inhibitor		Escitalopram
Fluoxetine				Fluoxetine; **Fluoxetine; *Fluoxetine***	Inhibitor; **Fluoxetine**	Fluoxetine, also strong inhibitor; **Fluoxetine; *Fluoxetine***		**Fluoxetine 3A4, 3A5**
Norfluoxetine				**Norfluoxetine**	**Norfluoxetine**	**Norfluoxetine**		Inhibitor; **Norfluoxetine 3A4,3A5**
Fluvoxamine	Fluvoxamine, also strong inhibitor; *Fluvoxamine*			Inhibitor	Inhibitor	Fluvoxamine; *Fluvoxamine*		Inhibitor
Paroxetine				Inhibitor		Paroxetine, also strong inhibitor; *Paroxetine*		*3A4 Paroxetine*
Sertraline				Inhibitor	*Sertraline*	moderate inhibitor		*3A4 Sertraline*
**Antidepressants: SNRIs**								
Desvenlafaxine					Desvenlafaxine	**Desvenlafaxine**		**Desvenlafaxine 3A4**
Duloxetine	Duloxetine; *Duloxetine*					Duloxetine, moderate inhibitor; *Duloxetine*		
Venlafaxine				Venlafaxine	Venlafaxine; **Venlafaxine**	Venlafaxine; **Venlafaxine**; *Venlafaxine*		Venlafaxine; **Venlafaxine 3A4**
**Antipsychotics: First-Generation**								
Chlorpromazine						Chlorpromazine, also inhibitor		
Haloperidol)	Haloperidol; *Haloperidol*					Haloperidol, also inhibitor; *Haloperidol*		Haloperidol; *3A4 Haloperidol*
Perphenazine						Perphenazine, also inhibitor		
**Second-Generation**								
Aripiprazole						Aripiprazole; *Aripiprazole*		Aripiprazole, *3A4 Aripiprazole*
Brexpiprazole						Brexpiprazole		Brexpiprazole
Cariprazine						Cariprazine		Cariprazine
Clozapine	Clozapine; *Clozapine*					Clozapine; *Clozapine*		
Olanzapine	**Olanzapine;** Olanzapine; *Olanzapine*			**Olanzapine**	**Olanzapine**	**Olanzapine;** *Olanzapine*		**Olanzapine 3A4, 3A5**
Quetiapine								Quetiapine; *3A4 Quetiapine*
Risperidone						Risperidone; *Risperidone*		Risperidone; *3A4 Risperidone*
Ziprasidone								Ziprasidone
**Barbiturates**								Barbiturates; Inducers
Butalbital (also **CYP2 A6**)	**Butalbital**		**Butalbital**	**Butalbital**			**Butalbital**	**Butalbital 3A4**
Hexobarbital					Hexobarbital			
Mephobarbital					Mephobarbital			
Phenobarbital/one		inducer		Inducer; *Phenobarbital*	Phenobarbital; *Phenobarbital*			Inducer
Secobarbital				Secobarbital				
**Benzodiazepines: short- and intermediate-acting:**								
Alprazolam								Alprazolam, *3A4 Alprazolam*
Diazepam	*Diazepam*			*Diazepam*	Diazepam; Diazepam; *Diazepam*			Diazepam
**Nonbenzodiazepine, benzodiazepine receptor agonist hypnotics ^a^**								
Zaleplon								Zaleplon
Zolpidem								Zolpidem
**Antiepileptics**								
Carbamazepine	Carbamazepine, Inducer	Carbamazepine, **Carbamazepine**	**Carbamazepine**	Inducer	Inducer, **Carbamazepine**			Carbamazepine, also inducer; **Carbamazepine 3A4,3A5,3A7**; *3A4 Carbamazepine*
Phenobarbitone		Inducer		Inducer	Phenobarbitone;			Inducer
Phenytoin	**Phenytoin**	Inducer		Phenytoin; **Phenytoin;** *Phenytoin*	**Phenytoin**; *Phenytoin*		**Phenytoin**	**Phenytoin 3A4;** Phenytoin, also inducer
Primidone					Primidone			
Topiramate					Inhibitor			
**Skeletal muscle relaxants**								
Carisoprodol					Carisoprodol	**Carisoprodol**		**Carisoprodol 3A4**
Chlorzoxazone							Chlorzoxazone	
Cyclobenzaprine	Cyclobenzaprine; *Cyclobenzaprine*					*Cyclobenzaprine*		
Methocarbamol			**Methocarbamol**	**Methocarbamol**	**Methocarbamol**			**Methocarbamol 3A4**
Orphenadrine, also CYP2A6	**Orphenadrine**		**Orphenadrine**	**Orphenadrine**			**Orphenadrine**	**Orphenadrine 3A4**
**Acetylcholinesterase inhibitors (AChEIs)**								
Donepezil						Donepezil; *Donepezil*		*3A4 Donepezil*
**Antiemetics**								
Metoclopramide						Metoclopramide, also inhibitor		
Promethazine						Promethazine, also inhibitor		
**STOPP renal system drugs**								
	**1A2**	**2B6**	**2C8**	**2C9**	**2C19**	**2D6**	**2E1**	**3A457**
Apixaban								*3A4 Apixaban*
Colchicine								*3A4 Colchicine*
Rivaroxaban								*3A4 Rivaroxaban*
For NSAIDs, please see musculoskeletal criteria								
**STOPP gastrointestinal system drugs**								
	**1A2**	**2B6**	**2C8**	**2C9**	**2C19**	**2D6**	**2E1**	**3A457**
Verapamil	Verapamil; Verapamil							Verapamil, also moderate inhibitor; *3A4 Verapamil*
Tolterodine								*3A4 Tolterodine*
**Anticholinergics: First-generation antihistamines**								
Chlorpheniramine						Chlorpheniramine, also inhibitor		Chlorpheniramine
Clemastine						inhibitor		
Diphenhydramine	*Diphenhydramine*		**Diphenhydramine**	**Diphenhydramine;** *Diphenhydramine*	**Diphenhydramine**; *Diphenhydramine*	*Diphenhydramine;* inhibitor		**Diphenhydramine 3A4**
Hydroxyzine						inhibitor		
Promethazine						**Promethazine,** also inhibitor Promethazine		**Promethazine**
**Proton-pump inhibitors**					*Proton-pump inhibitors*			*3A4 Proton-pump inhibitors*
Esomeprazole	Inducer				Esomeprazole, also strong inhibitor			Esomeprazole, also weak inhibitor
Lansoprazole					Lansoprazole, also inhibitor			Lansoprazole
Omeprazole	Inducer			*Omeprazole*	Omeprazole, also strong inhibitor			Omeprazole, also weak inhibitor
Pantoprazole					Pantoprazole, also weak inhibitor			Pantoprazole, also weak inhibitor
For opioids, see central nervous system drugs								
**STOPP respiratory system drugs**								
	**1A2**	**2B6**	**2C8**	**2C9**	**2C19**	**2D6**	**2E1**	**3A457**
Theophylline, also **CYP 2A6**	**Theophylline;** Theophylline; *Theophylline*		**Theophylline**	**Theophylline**			**Theophylline;** Theophylline	**Theophylline 3A4**
**Corticosteroids ^b^**								
Dexamethasone						Inducer		Dexamethasone; *3A4 Dexamethasone*
Hydrocortisone								Hydrocortisone
Prednisone					Inducer			Prednisone 3A4
For benzodiazepines, see central nervous system criteria								
**STOPP musculoskeletal drugs**								
	**1A2**	**2B6**	**2C8**	**2C9**	**2C19**	**2D6**	**2E1**	**3A457**
Colchicine								*3A4 Colchicine*
Aspirin >325 mg/d **CYP 2J2, 2U1, 4A11, and 4F8**		**Aspirin**	**Aspirin**				**Aspirin**	
**Non-cyclooxygenase-selective NSAIDs**								
Diclofenac, **also CYP 2J2, 2U1, 4A11, and 4F8**		**Diclofenac**	**Diclofenac**	Diclofenac; *Diclofenac*			**Diclofenac**	
Diflunisal, **also CYP 2J2, 2U1, 4A11, and 4F8**		**Diflunisal**	**Diflunisal**				**Diflunisal**	
Etodolac, **also CYP 2J2, 2U1, 4A11, and 4F8**		**Etodolac**	**Etodolac**				**Etodolac**	
Flurbiprofen, **also CYP 2J2, 2U1, 4A11, and 4F8**		**Flurbiprofen**	**Flurbiprofen**				**Flurbiprofen**	
Ibuprofen, **also CYP 2J2, 2U1, 4A11, and 4F8**		**Ibuprofen**	**Ibuprofen**	Ibuprofen; **Ibuprofen;** *Ibuprofen*	**Ibuprofen**		**Ibuprofen**	**Ibuprofen 3A4**
Indomethacin, also **CYP 2J2, 2U1, 4A11, and 4F8**		**Indomethacin**	**Indomethacin**	*Indomethacin*	Indomethacin, also inhibitor; *Indomethacin*		**Indomethacin**	
Ketoprofen, also **CYP 2J2, 2U1, 4A11, and 4F8**		**Ketoprofen**	**Ketoprofen**				**Ketoprofen**	
Nabumetone, also **CYP 2J2, 2U1, 4A11, and 4F8)**	Nabumetone	Nabumetone	**Nabumetone**				**Nabumetone**	
Naproxen, also **CYP 2J2, 2U1, 4A11, and 4F8**	Naproxen	**Naproxen**	**Naproxen**	Naproxen; *Naproxen*			**Naproxen**	
Piroxicam, **also CYP 2J2, 2U1, 4A11, and 4F8**		**Piroxicam**	**Piroxicam**	Piroxicam			**Piroxicam**	
Sulindac, also **CYP 2J2, 2U1, 4A11, and 4F8**		**Sulindac**	**Sulindac**				**Sulindac**	
Tolmetin, **also CYP 2J2, 2U1, 4A11, and 4F8**		**Tolmetin**	**Tolmetin**				**Tolmetin**	
COX-2 inhibitors								
Celecoxib, **also CYP 2J2, 2U1, 4A11, and 4F8**		**Celecoxib**	**Celecoxib**	Celecoxib; **Celecoxib**; *Celecoxib*		Inhibitor **Celecoxib**	**Celecoxib**	**Celecoxib 3A4**
Meloxicam, **YP 2J2, 2U1, 4A11, and 4F8**		**Meloxicam**	**Meloxicam**	Meloxicam			**Meloxicam**	
**Corticosteroids ^b^**								
Dexamethasone						Inducer		Dexamethasone; *3A4 Dexamethasone*
Hydrocortisone								Hydrocortisone
Prednisone					Inducer			**Prednisone 3A4**
**STOPP Urogenital system drugs**								
	**1A2**	**2B6**	**2C8**	**2C9**	**2C19**	**2D6**	**2E1**	**3A457**
Tamsulosin								*3A4 Tamsulosin*
**Mixed alpha-1 and beta antagonists**								
Labetalol					Labetalol			
Carvedilol				*Carvedilol*		Carvedilol; *Carvedilol*		
**STOPP Endocrine system drug**								
	**1A2**	**2B6**	**2C8**	**2C9**	**2C19**	**2D6**	**2E1**	**3A457**
**Sulfonylureas, long-acting**								
Glimepiride				Glimepiride; *Glimepiride*				
Glyburide				Glyburide; *Glyburide*				
**Thiazolidinediones**			Inhibitors					
Pioglitazone			**Pioglitazone** inhibitor	**Pioglitazone**				Pioglitazone, also inducer
Rosiglitazone			**Rosiglitazone***;* inhibitor	Rosiglitazone; **Rosiglitazone**; *Rosiglitazone*				
Troglitazone			Inhibitor					Troglitazone
**B-blockers**								
Bisoprolol						*Bisoprolol*		
Carvedilol (mixed alpha-1 and B-blocker)				*Carvedilol*		Carvedilol; *Carvedilol*		
Labetalol					Labetalol			
Metoprolol						Metoprolol; *Metoprolol*		
Propranolol	Propranolol; *Propranolol*				*Propranolol*	*Propranolol*		
Timolol						Timolol; *Timolol*		
**Hormones**								
Estrogens ^c^		**Estrogen**				**Estrogen**		**Estrogen 3A4, 3A5**
Testosterone (CYP11B1, 19A1)								Testosterone
**STOPP Drugs increasing risk of falls**								
	**1A2**	**2B6**	**2C8**	**2C9**	**2C19**	**2D6**	**2E1**	**3A457**
**Benzodiazepines: short- and intermediate-acting:**								
Alprazolam								Alprazolam, *3A4 Alprazolam*
Diazepam	*Diazepam*			*Diazepam*	Diazepam; Diazepam; *Diazepam*			Diazepam
**Nonbenzodiazepine, benzodiazepine receptor agonist hypnotics ^a^**								
Zaleplon								Zaleplon
Zolpidem								Zolpidem
**Antipsychotics: First-Generation**								
Chlorpromazine						Chlorpromazine, also inhibitor		
Haloperidol)	Haloperidol; *Haloperidol*					Haloperidol, also inhibitor; *Haloperidol*		Haloperidol; *3A4 Haloperidol*
Perphenazine						Perphenazine, also inhibitor		
**Antipsychotics: Second-Generation**								
Aripiprazole						Aripiprazole; *Aripiprazole*		Aripiprazole, *3A4 Aripiprazole*
Brexpiprazole						Brexpiprazole		Brexpiprazole
Cariprazine						Cariprazine		Cariprazine
Clozapine	Clozapine; *Clozapine*					Clozapine; *Clozapine*		
Olanzapine	**Olanzapine;** Olanzapine; *Olanzapine*			**Olanzapine**	**Olanzapine**	**Olanzapine;** *Olanzapine*		**Olanzapine 3A4, 3A5**
Quetiapine								Quetiapine; *3A4 Quetiapine*
Risperidone						Risperidone; *Risperidone*		Risperidone; *3A4 Risperidone*
Ziprasidone								Ziprasidone
**Vasodilators: B-blocker**								
Bisoprolol						*Bisoprolol*		
Carvedilol				*Carvedilol*		Carvedilol; *Carvedilol*		
Labetalol					Labetalol			
Metoprolol						Metoprolol; *Metoprolol*		
Propranolol	Propranolol; *Propranolol*				*Propranolol*	*Propranolol*		
Timolol						Timolol; *Timolol*		
**Vasodilators: calcium channel blockers**								
Amlodipine								Amlodipine; *3A4 Amlodipine*
Felodipine								Felodipine; *3A4 Felodipine*
Nifedipine								Nifedipine^e^ *3A4 Nifedipine*
Diltiazem								Diltiazem, also moderate inhibitor; *3A4* Diltiazem
Verapamil	Verapamil; Verapamil							Verapamil, also moderate inhibitor; *3A4 Verapamil*
**STOPP Analgesic drugs**								
	**1A2**	**2B6**	**2C8**	**2C9**	**2C19**	**2D6**	**2E1**	**3A457**
**Opioids**								
Codeine						Codeine; *Codeine*		Codeine 3A4
Fentanyl, Fentanil								Fentanyl, *3A4 Fentanyl*
Meperidine		Meperidine						
Morphine						**Morphine**		**Morphine**
Oxycodone						Oxycodone; *Oxycodone*		*3A4 Oxycodone*
Tramadol		Tramadol				Tramadol; *Tramadol*		Tramadol

Notes: ^a^ “Z-drugs”; ^b^ excludes ophthalmic; ^c^ excludes intravaginal. The Flockhart tables [26] define a moderate inhibitor as one that causes a >2-fold increase in the plasma AUC values or 50–80% decrease in clearance. A weak inhibitor is one that causes a >1.25-fold but <2-fold increase in the plasma AUC values or a 20–50% decrease in clearance.

**Table 5:** American Geriatric Society 2019 criteria for Potentially Inappropriate Medication Use in older adults for which the P450 cytochrome isoform is known. Sources: **Bold print** = DrugBank [25]; Regular print = Flockhart Table [26]; *Italic Print* = Rx Files. 12th ed. [27]. If a medication name is given it indicates it is a substrate metabolized by the P450 cytochromes in the columns listed. If a medication is also an inducer of individual P450 cytochromes the word inducer is used. If a medication is an inhibitor of individual P450 cytochromes the word inhibitor is used. (Inhibitors increase levels of medications metabolized by the same isoform. Inducers decrease levels of medications metabolized by the same isoform). However, if they metabolize a prodrug (e.g., codeine) the result is higher levels of its metabolites (e.g., morphine).

AGS Medications to Avoid: Anticholinergics								
	1A2	2B6	2C8	2C9	2C19	2D6	2E1	3A457
**Anticholinergics: first-generation antihistamines**								
Chlorpheniramine						Chlorpheniramine, also inhibitor		Chlorpheniramine
Clemastine						inhibitor		
Diphenhydramine	*Diphenhydramine*		**Diphenhydramine**	**Diphenhydramine**; *Diphenhydramine*	**Diphenhydramine**; *Diphenhydramine*	*Diphenhydramine;* inhibitor		**Diphenhydramine 3A4**
Hydroxyzine						inhibitor		
Promethazine						Promethazine, also inhibitor **Promethazine**		**Promethazine**
Orphenadrine, also CYP2A6	**Orphenadrine**		**Orphenadrine**	**Orphenadrine**			**Orphenadrine**	**Orphenadrine 3A4**
**AGS Medications to avoid: Cardiovascular system**								
	**1A2**	**2B6**	**2C8**	**2C9**	**2C19**	**2D6**	**2E1**	**3A457**
Amiodarone	Inhibitor			Moderate inhibitor		Weak inhibitor		*3A4 Amiodarone*; Inhibitor
Carvedilol				*Carvedilol*		Carvedilol; *Carvedilol*		
Labetalol					Labetalol			
Nifedipine								Nifedipine *3A4 Nifedipine*
**AGS Medications to avoid: Central nervous system**								
	**1A2**	**2B6**	**2C8**	**2C9**	**2C19**	**2D6**	**2E1**	**3A457**
**Antidepressants: tricyclics**								
Amitriptyline	Amitriptyline; *Amitriptyline*			Amitriptyline; *Amitriptyline*	Amitriptyline; *Amitriptyline*	Amitriptyline; *Amitriptyline*		Amitriptyline; *3A4 Amitriptyline*
Clomipramine	Clomipramine; **Clomipramine**; *Clomipramine*				Clomipramine; **Clomipramine**; *Clomipramine*	Clomipramine, also inhibitor **Clomipramine**; *Clomipramine*		**Clomipramine**
Desipramine	**Desipramine**; *Desipramine*				**Desipramine**; *Desipramine*	Desipramine **Desipramine**;		**Desipramine 3A4**
Doxepin >6 mg/d	Doxepin; **Doxepin**			Doxepin; **Doxepin**; *Doxepin*	Doxepin; **Doxepin**; *Doxepin*	Doxepin, also inhibitor; **Doxepin**; *Doxepin*		Doxepin; **Doxepin 3A4**
Imipramine	Imipramine; **Imipramine**; *Imipramine*				Imipramine; **Imipramine**	Imipramine; **Imipramine**		**Imipramine 3A4**
Nortriptyline						Nortriptyline; *Nortriptyline*		
**Antidepressants: SSRIs**								
Citalopram	Weak inhibitor				Citalopram, also weak inhibitor; **Citalopram;** *Citalopram*	Citalopram, also weak inhibitor; **Citalopram**		Citalopram; **Citalopram 3A7**; *3A4 Citalopram*
Escitalopram					Escitalopram; *Escitalopram*	Escitalopram, also weak inhibitor		Escitalopram
Fluoxetine				Fluoxetine; **Fluoxetine; *Fluoxetine***	inhibitor; **Fluoxetine**	Fluoxetine, also strong inhibitor; **Fluoxetine; *Fluoxetine***		**Fluoxetine 3A4, 3A5**
Norfluoxetine				**Norfluoxetine**	**Norfluoxetine**	**Norfluoxetine**		Inhibitor; **Norfluoxetine 3A4,3A5**
Fluvoxamine	Fluvoxamine, also strong inhibitor; *Fluvoxamine*			Inhibitor	Inhibitor	Fluvoxamine; *Fluvoxamine*		Inhibitor
Paroxetine				Inhibitor		Paroxetine, also strong inhibitor; *Paroxetine*		*3A4 Paroxetine*
Sertraline				Inhibitor	*Sertraline*	moderate inhibitor		*3A4 Sertraline*
**Antidepressants: SNRIs**					Desvenlafaxine	**Desvenlafaxine**		**Desvenlafaxine 3A4**
Desvenlafaxine								
Duloxetine	Duloxetine; *Duloxetine*					Duloxetine, moderate inhibitor; *Duloxetine*		
Venlafaxine				Venlafaxine	Venlafaxine; **Venlafaxine**	Venlafaxine; **Venlafaxine;** *Venlafaxine*		Venlafaxine; **Venlafaxine 3A4**
**Antipsychotics:** **First-Generation**								
Chlorpromazine						Chlorpromazine, also inhibitor		
Haloperidol)	Haloperidol; *Haloperidol*					Haloperidol, also inhibitor; *Haloperidol*		Haloperidol; *3A4 Haloperidol*
Perphenazine						Perphenazine, also inhibitor		
**Second-Generation**								
Aripiprazole						Aripiprazole; *Aripiprazole*		Aripiprazole, *3A4 Aripiprazole*
Brexpiprazole						Brexpiprazole		Brexpiprazole
Cariprazine						Cariprazine		Cariprazine
Clozapine	Clozapine; *Clozapine*					Clozapine; *Clozapine*		
Olanzapine	**Olanzapine;** Olanzapine; *Olanzapine*			**Olanzapine**	**Olanzapine**	**Olanzapine;** *Olanzapine*		**Olanzapine 3A4, 3A5**
Quetiapine								Quetiapine; *3A4 Quetiapine*
Risperidone						Risperidone; *Risperidone*		Risperidone; *3A4 Risperidone*
Ziprasidone								Ziprasidone
**Barbiturates**								Barbiturates; Inducers
Butalbital (also **CYP2 A6**)	**Butalbital**		**Butalbital**	**Butalbital**			**Butalbital**	**Butalbital 3A4**
Hexobarbital					Hexobarbital			
Mephobarbital					Mephobarbital			
Phenobarbital/one		inducer		Inducer; *Phenobarbital*	Phenobarbital; *Phenobarbital*			Inducer
Secobarbital				Secobarbital				
**Benzodiazepines: short- and intermediate-acting**:								
Alprazolam								Alprazolam, *3A4 Alprazolam*
Diazepam	*Diazepam*			*Diazepam*	Diazepam; Diazepam; *Diazepam*			Diazepam
**Nonbenzodiazepine, benzodiazepine receptor agonist hypnotics ^a^**								
Zaleplon								Zaleplon
Zolpidem								Zolpidem
**AGS Medications to avoid: Endocrine system**								
	1A2	2B6	2C8	2C9	2C19	2D6	2E1	3A457
Testosterone (CYP11B1, 19A1)								Testosterone
Estrogens ^c^		**Estrogen**				**Estrogen**		**Estrogen 3A4, 3A5**
Insulin	Inducer							
**Sulfonylureas, long-acting**								
Chlorpropamide								
Glimepiride				Glimepiride; *Glimepiride*				
Glyburide				Glyburide; *Glyburide*				
**AGS Medications to avoid: Gastrointestinal system**								
	1A2	2B6	2C8	2C9	2C19	2D6	2E1	3A457
Metoclopramide						Metoclopramide		
**Proton-pump inhibitors**					*Proton-pump inhibitors*			*3A4 Proton-pump inhibitors*
Esomeprazole	Inducer				Esomeprazole, also strong inhibitor			Esomeprazole, also weak inhibitor
Lansoprazole					Lansoprazole, also inhibitor			Lansoprazole
Omeprazole	Inducer			*Omeprazole*	Omeprazole, also strong inhibitor			Omeprazole, also weak inhibitor
Pantoprazole					Pantoprazole, also weak inhibitor			Pantoprazole, also weak inhibitor
**AGS Medications to avoid: Analgesic drugs**								
	1A2	2B6	2C8	2C9	2C19	2D6	2E1	3A457
Meperidine		Meperidine						
**Skeletal muscle relaxants**								
Carisoprodol					Carisoprodol	**Carisoprodol**		**Carisoprodol 3A4**
Chlorzoxazone							**Chlorzoxazone**	
Cyclobenzaprine	Cyclobenzaprine; *Cyclobenzaprine*					*Cyclobenzaprine*		
Methocarbamol			**Methocarbamol**	**Methocarbamol**	**Methocarbamol**			**Methocarbamol 3A4**
Orphenadrine, also CYP2A6	**Orphenadrine**		**Orphenadrine**	**Orphenadrine**			**Orphenadrine**	**Orphenadrine 3A4**
**Non-cyclooxygenase-selective NSAIDs**								
Diclofenac, **also CYP 2J2, 2U1, 4A11, and 4F8**		**Diclofenac**	**Diclofenac**	Diclofenac; *Diclofenac*			**Diclofenac**	
Diflunisal, **also CYP 2J2, 2U1, 4A11, and 4F8**		**Diflunisal**	**Diflunisal**				**Diflunisal**	
Etodolac, **also CYP 2J2, 2U1, 4A11, and 4F8**		**Etodolac**	**Etodolac**				**Etodolac**	
Flurbiprofen, **also CYP 2J2, 2U1, 4A11, and 4F8**		**Flurbiprofen**	**Flurbiprofen**				**Flurbiprofen**	
Ibuprofen, **also CYP 2J2, 2U1, 4A11, and 4F8**		**Ibuprofen**	**Ibuprofen**	Ibuprofen; **Ibuprofen**; *Ibuprofen*	**Ibuprofen**		**Ibuprofen**	**Ibuprofen 3A4**
Indomethacin, **CYP 2J2, 2U1, 4A11, and 4F8**		**Indomethacin**	**Indomethacin**	*Indomethacin*	Indomethacin, also inhibitor; *Indomethacin*		**Indomethacin**	
Ketoprofen, **also CYP 2J2, 2U1, 4A11, and 4F8**		**Ketoprofen**	**Ketoprofen**				**Ketoprofen**	
Nabumetone, **CYP 2J2, 2U1, 4A11, and 4F8)**	Nabumetone	Nabumetone	**Nabumetone**				**Nabumetone**	
Naproxen, **CYP 2J2, 2U1, 4A11, and 4F8**	Naproxen	**Naproxen**	**Naproxen**	Naproxen; *Naproxen*			**Naproxen**	
Piroxicam, **CYP 2J2, 2U1, 4A11, and 4F8**		**Piroxicam**	**Piroxicam**	Piroxicam			**Piroxicam**	
Sulindac, **also CYP 2J2, 2U1, 4A11, and 4F8**		**Sulindac**	**Sulindac**				**Sulindac**	
Tolmetin, **also CYP 2J2, 2U1, 4A11, and 4F8**		**Tolmetin**	**Tolmetin**				**Tolmetin**	
**AGS PIMS: exacerbations of interactions of drugs and diseases: heart failure**								
	1A2	2B6	2C8	2C9	2C19	2D6	2E1	3A457
Cilostazol								Cilostazol
Diltiazem								Diltiazem, also moderate inhibitor; *3A4* Diltiazem
Meperidine		Meperidine						
Verapamil	Verapamil; *Verapamil*							Verapamil, also moderate inhibitor; *3A4 Verapamil*
NSAIDs and Cox-2: see STOPP Musculoskeletal drugs								
**Thiazolidinediones**			Inhibitors					
Pioglitazone			**Pioglitazone** inhibitor	**Pioglitazone**				Pioglitazone, also inducer
Rosiglitazone			**Rosiglitazone***;* inhibitor	Rosiglitazone; **Rosiglitazone**; *Rosiglitazone*				
Troglitazone			Inhibitor					Troglitazone
**AGS PIMs: exacerbations of interactions of drugs and diseases: syncope**								
	**1A2**	**2B6**	**2C8**	**2C9**	**2C19**	**2D6**	**2E1**	**3A457**
Chlorpromazine						Chlorpromazine, also inhibitor		
**Acetylcholinesterase inhibitors (AChEIs)**								
Donepezil						Donepezil; *Donepezil*		*3A4 Donepezil*
**Mixed alpha-1 and B-blockers**								
Carvedilol				*Carvedilol*		Carvedilol; *Carvedilol*		
Labetalol					Labetalol			
**AGS PIMs: exacerbations of interactions of drugs and diseases: delirium, dementia, cognitive impairment, falls, and Parkinsonism**								
	**1A2**	**2B6**	**2C8**	**2C9**	**2C19**	**2D6**	**2E1**	**3A457**
Anticholinergics see Table								
Antipsychotics, benzodiazepines anticonvulsants, antiemetics: see STOPP Central nervous system								
**H-2 receptor antagonists**								
Cimetidine	Weak inhibitor		**Cimetidine**	**Cimetidine**	**Cimetidine** inhibitor	Weak inhibitor		**Cimetidine**; weak inhibitor
Ranitidine						Inhibitor		
**AGS PIMs: exacerbations of interactions of drugs and diseases: gastric/duodenal ulcers**								
**For aspirin and non-Cox-2 selective NSAIDs see STOPP musculoskeletal list**								
**AGS PIMs: exacerbations of interactions of drugs and diseases: kidney and urinary tract disorders**								
	**1A2**	**2B6**	**2C8**	**2C9**	**2C19**	**2sD6**	**2E1**	**3A457**
Estrogens ^c^		**Estrogen**				**Estrogen**		**Estrogen 3A4, 3A5**
Tamsulosin								*3A4 Tamsulosin*
**Mixed alpha-1 and beta antagonists**								
Labetalol					Labetalol			
Carvedilol				*Carvedilol*		Carvedilol; *Carvedilol*		
**AGS medications to be used with caution**								
	**1A2**	**2B6**	**2C8**	**2C9**	**2C19**	**2D6**	**2E1**	**3A457**
Aspirin >325 mg/d **CYP 2J2, 2U1, 4A11, and 4F8**		**Aspirin**	**Aspirin**				**Aspirin**	
Antipsychotics, SSRIs, SNRIs, tricyclics see STOPP central nervous system								
Trimethoprim			Moderate inhibitor					
Sulfamethoxazole				Inhibitor; *Sulfamethoxazole*				
Dextromethorphan						Dextromethorphan; *Dextromethorphan*		Dextromethorphan
Quinidine						Strong inhibitor		Quinidine
Diuretics								
Eplerenone								Eplerenone
Triamterene	Triamterene							
Torsemide			Torsemide	Torsemide				
Triamterene	Triamterene							
**AGS PIMS avoid these drug–drug interactions**								
	**1A2**	**2B6**	**2C8**	**2C9**	**2C19**	**2D6**	**2E1**	**3A457**
**Combination 5A**	**Renin–angiotensin inhibitors and angiotensin II receptor blockers** with triamterene or amiloride or another renin–angiotensin **inhibitor**							
Triamterene	Triamterene							
**Angiotensin-II receptor blockers**								
Irbesartan				Irbesartan; *Irbesartan*				
Losartan				Losartan; *Losartan*				
Valsartan				*Valsartan*				
**Combination 5B**	Benzodiazepines with opioids (see STOPP CNS list in Table 2)							
**Combination 5C**	Opioids (see STOPP CNS list in Table 2) with gabapentin or pregabalin							
**Combination 5D**	≥2 anticholinergics							
**Combination 5E**	≥3 CNS active drugs (TCAs or SSRIs or SNRIs or antiepileptics or benzodiazepines or hypnotic Z-drugs or opioids (see STOPP CNS list in Table 2))							
**Combination 5F**	Systemic corticosteroids ^b^ with opioids (see STOPP CNS list in Table 2)							
**Combination 5G**	Lithium with ACE inhibitor							
**Combination 5H**	Lithium with Loop diuretic							
**Loop diuretics**								
Eplerenone								Eplerenone
Torsemide			Torsemide	Torsemide				
Triamterene	Triamterene							
**Combination 5I**	Peripheral alpha-blocker with loop diuretic							
**Mixed alpha-1 and beta antagonists**								
Labetalol					Labetalol			
Carvedilol				*Carvedilol*		Carvedilol; *Carvedilol*		
**Loop diuretics**								
Eplerenone								Eplerenone
Torsemide			Torsemide	Torsemide				
Triamterene	Triamterene							
**Combination 5J**	Phenytoin with Trimethoprim-sulfamethoxazole							
Phenytoin	**Phenytoin**	Inducer		Phenytoin; **Phenytoin;** *Phenytoin*	**Phenytoin;** *Phenytoin*		**Phenytoin**	**Phenytoin 3A4;** Phenytoin, also inducer
Trimethoprim			Moderate inhibitor					
Sulfamethoxazole				Inhibitor; *Sulfamethoxazole*				
**Combination 5K**	Theophylline with cimetidine							
Theophylline, also **CYP 2A6**	**Theophylline**; Theophylline; *Theophylline*		**Theophylline**	**Theophylline**			**Theophylline;** Theophylline	**Theophylline 3A4**
Cimetidine	Weak inhibitor		**Cimetidine**	**Cimetidine**	**Cimetidine** inhibitor	Weak inhibitor		**Cimetidine**; weak inhibitor
**Combination 5L**	Theophylline with ciprofloxacin							
Theophylline, also **CYP 2A6**	**Theophylline;** Theophylline; *Theophylline*		**Theophylline**	**Theophylline**			**Theophylline;** Theophylline	**Theophylline 3A4**
Ciprofloxacin	Strong inhibitor							Inhibitor
**Combination 5M**	Warfarin with amiodarone							
Warfarin	Warfarin; *Warfarin*			s-Warfarin; *Warfarin*	r-Warfarin; *Warfarin*			*3A4 Warfarin*
Amiodarone	Inhibitor			Moderate inhibitor		Weak inhibitor		*3A4 Amiodarone*; Inhibitor
**Combination 5N**	Warfarin with ciprofloxacin							
Warfarin	Warfarin; *Warfarin*			s-Warfarin; *Warfarin*	r-Warfarin; *Warfarin*			*3A4 Warfarin*
Ciprofloxacin	Strong inhibitor							Inhibitor
**Combination 5N**	Warfarin with macrolide							
Warfarin	Warfarin; *Warfarin*			s-Warfarin; *Warfarin*	r-Warfarin; *Warfarin*			*3A4 Warfarin*
Clarithromycin								Clarithromycin, also strong inhibitor; *3A4 Clarithromycin*
Erythromycin								Erythromycin, also moderate inhibitor; *3A4 Erythromycin*
Telithromycin								Telithromycin, also strong inhibitor
**Combination 5O**	Warfarin with Trimethoprim-sulfamethoxazole							
Warfarin	Warfarin; *Warfarin*			s-Warfarin; *Warfarin*	r-Warfarin; *Warfarin*			*3A4 Warfarin*
Trimethoprim			Moderate inhibitor					
Sulfamethoxazole				Inhibitor; *Sulfamethoxazole*				
**Combination 5Q**			Warfarin with NSAID					
Warfarin	Warfarin; *Warfarin*			s-Warfarin; *Warfarin*	r-Warfarin; *Warfarin*			*3A4 Warfarin*
**Non-cyclooxygenase-selective NSAIDs**								
Diclofenac, **also CYP 2J2, 2U1, 4A11, and 4F8**		**Diclofenac**	**Diclofenac**	Diclofenac; *Diclofenac*			**Diclofenac**	
Diflunisal, **also CYP 2J2, 2U1, 4A11, and 4F8**		**Diflunisal**	**Diflunisal**				**Diflunisal**	
Etodolac, **also CYP 2J2, 2U1, 4A11, and 4F8**		**Etodolac**	**Etodolac**				**Etodolac**	
Flurbiprofen, **also CYP 2J2, 2U1, 4A11, and 4F8**		**Flurbiprofen**	**Flurbiprofen**				**Flurbiprofen**	
Ibuprofen, **also CYP 2J2, 2U1, 4A11, and 4F8**		**Ibuprofen**	**Ibuprofen**	**Ibuprofen**; Ibuprofen; *Ibuprofen*	**Ibuprofen**		**Ibuprofen**	**Ibuprofen 3A4**
Indomethacin, **CYP 2J2, 2U1, 4A11, and 4F8**		**Indomethacin**	**Indomethacin**	*Indomethacin*	Indomethacin, also inhibitor; *Indomethacin*		**Indomethacin**	
Ketoprofen, **also CYP 2J2, 2U1, 4A11, and 4F8**		**Ketoprofen**	**Ketoprofen**				**Ketoprofen**	
Nabumetone, **CYP 2J2, 2U1, 4A11, and 4F8)**	Nabumetone	Nabumetone	**Nabumetone**				**Nabumetone**	
Naproxen, **CYP 2J2, 2U1, 4A11, and 4F8**	Naproxen	**Naproxen**	**Naproxen**	Naproxen; *Naproxen*			**Naproxen**	
Piroxicam, **CYP 2J2, 2U1, 4A11, and 4F8**		**Piroxicam**	**Piroxicam**	Piroxicam			**Piroxicam**	
Sulindac, also **CYP 2J2, 2U1, 4A11, and 4F8**		**Sulindac**	**Sulindac**				**Sulindac**	
Tolmetin, also **CYP 2J2, 2U1, 4A11, and 4F8**		**Tolmetin**	**Tolmetin**				**Tolmetin**	
**AGS PIMS to be avoided or dosage reduced with varying levels of kidney function**								
**AGS PIMS to be avoided or dosage reduced: anti-infectives**								
Ciprofloxacin	Strong inhibitor							Inhibitor
Trimethoprim			Moderate inhibitor					
Sulfamethoxazole				Inhibitor; *Sulfamethoxazole*				
**AGS PIMS to be avoided or dosage reduced: cardiovascular or hemostasis**								
Apixaban								*3A4 Apixaban*
Rivaroxaban								*3A4 Rivaroxaban*
Triamterene	Triamterene							
**AGS PIMS to be avoided or dosage reduced: central nervous system and analgesics**								
Duloxetine	Duloxetine; *Duloxetine*					Duloxetine, moderate inhibitor; *Duloxetine*		
Tramadol		Tramadol				Tramadol; *Tramadol*		Tramadol
**AGS PIMS to be avoided or dosage reduced: gastrointestinal**								
Cimetidine	Weak inhibitor		**Cimetidine**	**Cimetidine**	**Cimetidine** inhibitor	Weak inhibitor		**Cimetidine**; weak inhibitor
Ranitidine						Inhibitor		
**AGS PIMS to be avoided or dosage reduced: hyperuricemia**								
Colchicine								*3A4 Colchicine*
Probenecid				Inhibitor	Inhibitor			
**AGS PIMS with strong anticholinergic properties**								
**AGS PIMS with strong anticholinergic properties: antiarrhythmics**								
**AGS PIMS with strong anticholinergic properties: antidepressants**								
Amitriptyline	Amitriptyline; *Amitriptyline*			Amitriptyline; *Amitriptyline*	Amitriptyline; *Amitriptyline*	Amitriptyline; *Amitriptyline*		Amitriptyline; *3A4 Amitriptyline*
Clomipramine	Clomipramine; **Clomipramine**; *Clomipramine*				Clomipramine; **Clomipramine**; *Clomipramine*	Clomipramine, also inhibitor **Clomipramine**; *Clomipramine*		**Clomipramine**
Desipramine	**Desipramine**; *Desipramine*				**Desipramine**; *Desipramine*	Desipramine **Desipramine**;		**Desipramine 3A4**
Doxepin >6 mg/d	Doxepin; **Doxepin**			Doxepin; **Doxepin**; *Doxepin*	Doxepin; **Doxepin**; *Doxepin*	Doxepin, also inhibitor; **Doxepin**; *Doxepin*		Doxepin; **Doxepin 3A4**
Imipramine	Imipramine; **Imipramine**; *Imipramine*				Imipramine; **Imipramine**	Imipramine; **Imipramine**		**Imipramine 3A4**
Nortriptyline						Nortriptyline; *Nortriptyline*		
Paroxetine				Inhibitor		Paroxetine, also strong inhibitor; *Paroxetine*		*3A4 Paroxetine*
**AGS PIMS with strong anticholinergic properties: antiemetics**								
Promethazine						Promethazine, also inhibitor **Promethazine**		**Promethazine**
**AGS PIMS with strong anticholinergic properties: antihistamines (first-generation)**								
Chlorpheniramine						Chlorpheniramine, also inhibitor		Chlorpheniramine
Clemastine						inhibitor		
Diphenhydramine	*Diphenhydramine*		**Diphenhydramine**	**Diphenhydramine**; *Diphenhydramine*	**Diphenhydramine**; *Diphenhydramine*	*Diphenhydramine;* inhibitor		**Diphenhydramine 3A4**
Hydroxyzine						inhibitor		
Promethazine						Promethazine, also inhibitor **Promethazine**		**Promethazine**
**AGS PIMS with strong anticholinergic properties: antimuscarinics**								
Tolterodine								*3A4 Tolterodine*
**AGS PIMS with strong anticholinergic properties: anti-Parkinson’s medications**								
**AGS PIMS with strong anticholinergic properties: antipsychotics (Criterion 7G)**								
Chlorpromazine						Chlorpromazine, also inhibitor		
Clozapine	Clozapine; *Clozapine*					Clozapine; *Clozapine*		
Olanzapine	**Olanzapine;** Olanzapine; *Olanzapine*			**Olanzapine**	**Olanzapine**	**Olanzapine;** *Olanzapine*		**Olanzapine 3A4, 3A5**
Perphenazine						Perphenazine, also inhibitor		
**AGS PIMS with strong anticholinergic properties: antispasmodics**								
**AGS PIMS with strong anticholinergic properties: Skeletal muscle relaxants**								
Cyclobenzaprine	Cyclobenzaprine; *Cyclobenzaprine*					*Cyclobenzaprine*		
Orphenadrine, also CYP2A6	**Orphenadrine**		**Orphenadrine**	**Orphenadrine**			**Orphenadrine**	**Orphenadrine 3A4**

Notes: ^a^ “Z-drugs”; ^b^ excludes ophthalmic; ^c^ excludes intravaginal. The Flockhart tables [26] define a moderate inhibitor as one that causes a >2-fold increase in the plasma AUC values or 50–80% decrease in clearance. A Weak inhibitor is one that causes a >1.25-fold but <2-fold increase in the plasma AUC values or a 20–50% decrease in clearance.

**Table 6:** START 2015 criteria for Potential Prescribing Omissions in older adults for which the P450 cytochrome isoform is known. Sources: **Bold print** = DrugBank [25]; Regular print = Flockhart Table [26]; *Italic Print* = Rx Files. 12th ed. [27]. If a medication name is given it indicates it is a substrate metabolized by the P450 cytochromes in the columns listed. If a medication is also an inducer of individual P450 cytochromes the word inducer is used. If a medication is an inhibitor of individual P450 cytochromes the word inhibitor is used. (Inhibitors increase levels of medications metabolized by the same isoform. Inducers decrease levels of medications metabolized by the same isoform). However, if they metabolize a prodrug (e.g., codeine) the result is higher levels of its metabolites (e.g., morphine).

START Cardiovascular System (Criteria A1 to A8)								
START Cardiovascular A1: Vitamin K Antagonist or Thrombin/Factor Xa Inhibitor with Chronic Atrial Fibrillation								
	1A2	2B6	2C8	2C9	2C19	2D6	2E1	3A457
Apixaban								*3A4 Apixaban*
Rivaroxaban								*3A4 Rivaroxaban*
Warfarin	Warfarin; *Warfarin*			s-Warfarin; *Warfarin*	r-Warfarin; *Warfarin*			*3A4 Warfarin*
**START Cardiovascular A2: Aspirin with chronic atrial fibrillation and contraindicated Vitamin K antagonist or thrombin/factor Xa inhibitor**								
Aspirin >325 mg/d also **CYP 2J2, 2U1, 4A11, and 4F8**		**Aspirin**	**Aspirin**				**Aspirin**	
**START Cardiovascular A3: Antiplatelet therapy with coronary, cerebral, or peripheral vascular disease**								
Aspirin >325 mg/d also **CYP 2J2, 2U1, 4A11, and 4F8**		**Aspirin**	**Aspirin**				**Aspirin**	
Clopidogrel	*Clopidogrel*	**Clopidogrel** inhibitor		**Clopidogrel;** Clopidogrel, also inhibitor	**Clopidogrel;** Clopidogrel; *Clopidogrel*			**Clopidogrel 3A4,3A5** **Clopidogrel**
Dipyridamole, also **CYP 2J2, 2U1, 4A11, and 4F8**		**Dipyridamole**	**Dipyridamole**				**Dipyridamole**	
Ticagrelor								*3A4 Ticagrelor*
Ticlopidine	**Ticlopidine**; inhibitor	**Ticlopidine;** inhibitor		**Ticlopidine**	**Ticlopidine;** inhibitor	Inhibitor		**Ticlopidine 3A4, 3A5**
**START Cardiovascular A4: Antihypertensive therapy for hypertension**								
**Angiotensin-II receptor blockers**								
Irbesartan				Irbesartan; *Irbesartan*				
Losartan				Losartan; *Losartan*				
Valsartan				*Valsartan*				
**Mixed alpha-1 and beta antagonists**								
Labetalol					Labetalol			
Carvedilol				*Carvedilol*		Carvedilol; *Carvedilol*		
**Vasodilators: B-blocker**								
Bisoprolol						*Bisoprolol*		
Carvedilol				*Carvedilol*		Carvedilol; *Carvedilol*		
Labetalol					Labetalol			
Metoprolol						Metoprolol; *Metoprolol*		
Propranolol	Propranolol; *Propranolol*				*Propranolol*	*Propranolol*		
Timolol						Timolol; *Timolol*		
**Vasodilators: Calcium channel blockers**								
Amlodipine								Amlodipine; *3A4 Amlodipine*
Felodipine								Felodipine; *3A4 Felodipine*
Nifedipine								Nifedipine ^e^ *3A4 Nifedipine*
Diltiazem								Diltiazem, also moderate inhibitor; *3A4* Diltiazem
Verapamil	Verapamil; Verapamil							Verapamil, also moderate inhibitor; *3A4 Verapamil*
**START Cardiovascular A5: Statin with coronary, cerebral, or peripheral vascular disease**								
Atorvastatin								Atorvastatin; *3A4 Atorvastatin*
Simvastatin								Simvastatin; *3A4 Simvastatin*
Rosuvastatin				Rosuvastatin	Rosuvastatin			
**START Cardiovascular A6: ACE inhibitors with ischemic heart disease**								
**START Cardiovascular A7: beta-blocker with ischemic heart disease and A8: beta-blocker with stable systolic heart failure**								
Bisoprolol						*Bisoprolol*		
Carvedilol				*Carvedilol*		Carvedilol; *Carvedilol*		
Labetalol					Labetalol			
Metoprolol						Metoprolol; *Metoprolol*		
Propranolol	Propranolol; *Propranolol*				*Propranolol*	*Propranolol*		
Timolol						Timolol; *Timolol*		
**START Respiratory system (Criteria B1-B3)**								
Budesonide								*3A4 Budesonide*
Fluticasone								*3A4 Fluticasone*
**START: Central nervous system and eyes (criteria C1 to C6)**								
**START CNS C1: L-Dopa or dopamine agonist in Parkinson’s with functional impairment or disability**								
**START CNS C2: Non-TCA antidepressant with persistent major depressive disorder**								
**Antidepressants: SSRIs**								
Citalopram	Weak inhibitor				Citalopram, also weak inhibitor; **Citalopram;** *Citalopram*	Citalopram, also weak inhibitor; **Citalopram**		Citalopram; **Citalopram 3A7**; *3A4 Citalopram*
Escitalopram					Escitalopram; *Escitalopram*	Escitalopram, also weak inhibitor		Escitalopram
Fluoxetine				Fluoxetine; **Fluoxetine; *Fluoxetine***	inhibitor; **Fluoxetine**	Fluoxetine, also strong inhibitor; **Fluoxetine; *Fluoxetine***		**Fluoxetine 3A4, 3A5**
Norfluoxetine				**Norfluoxetine**	**Norfluoxetine**	**Norfluoxetine**		Inhibitor; **Norfluoxetine 3A4,3A5**
Fluvoxamine	Fluvoxamine, also strong inhibitor; *Fluvoxamine*			Inhibitor	Inhibitor	Fluvoxamine; *Fluvoxamine*		Inhibitor
Paroxetine				Inhibitor		Paroxetine, also strong inhibitor; *Paroxetine*		*3A4 Paroxetine*
Sertraline				Inhibitor	*Sertraline*	moderate inhibitor		*3A4 Sertraline*
**Antidepressants: SNRIs**					Desvenlafaxine	**Desvenlafaxine**		**Desvenlafaxine 3A4**
Desvenlafaxine								
Duloxetine	Duloxetine; *Duloxetine*					Duloxetine, moderate inhibitor; *Duloxetine*		
Venlafaxine				Venlafaxine	Venlafaxine; **Venlafaxine**	Venlafaxine; **Venlafaxine;** *Venlafaxine*		Venlafaxine; **Venlafaxine 3A4**
**START CNS C3: ACE inhibitor for mild–moderate Alzheimer’s or Lewy body dementia**								
**START CNS C4: Topical prostaglandin, prosamide, or beta-blocker for primary open-angle glaucoma**								
Timolol						Timolol; *Timolol*		
**START CNS C5: SSRI, SNRI, or pregabalin for persistent anxiety (See STOPP CNS list)**								
**START CNS C5: Dopamine agonist for restless legs syndrome**								
**START Gastrointestinal System D1, D2: Proton-pump inhibitor with severe gastroesophageal reflux disease or peptic stricture**								
Esomeprazole	Inducer				Esomeprazole, also strong inhibitor			Esomeprazole, also weak inhibitor
Lansoprazole					Lansoprazole, also inhibitor			Lansoprazole
Omeprazole	Inducer			*Omeprazole*	Omeprazole, also strong inhibitor			Omeprazole, also weak inhibitor
Pantoprazole					Pantoprazole, also weak inhibitor			Pantoprazole, also weak inhibitor
**START Musculoskeletal system (criteria E1 to E7)**								
**START Musculoskeletal system E1: Disease-modifying antirheumatic drug**								
**START Musculoskeletal system E2: Bisphosphonates, vitamin D_3_ and calcium with long-term systematic corticosteroids; E3 with osteoporosis/fracture; E4 with osteoporosis; E5 Vitamin D_3_ with falls or osteopenia**								
**START Musculoskeletal system E6: xanthine-oxidase inhibitor with gout**								
**START Musculoskeletal system E7: Folic acid supplementation with methotrexate**								
**START Endocrine system F: ACE inhibitor or ARB in diabetes with renal disease**								
**Angiotensin-II receptor blockers**								
Irbesartan				Irbesartan; *Irbesartan*				
Losartan				Losartan; *Losartan*				
Valsartan				*Valsartan*				
**START Urogenital G1: Alpha-1 receptor blockers with prostatism and no prostatectomy**								
**START Urogenital G2: 5-alpha reductase inhibitors with prostatism and no prostatectomy**								
**START Analgesic drugs H1, H2:**								
Fentanyl, Fentanil								Fentanyl, *3A4 Fentanyl*
Meperidine		Meperidine						
Oxycodone						Oxycodone; *Oxycodone*		*3A4 Oxycodone*

Notes: The Flockhart [26] tables define a moderate inhibitor as one that causes a >2-fold increase in the plasma AUC values or 50–80% decrease in clearance. A Weak inhibitor is one that causes a >1.25-fold but <2-fold increase in the plasma AUC values or a 20–50% decrease in clearance.

## Supplementary Materials

The following are available online at https://www.mdpi.com/2308-3417/5/4/64/s1, Table S1: Genetic influences on efflux pumps and organic anion-transporting polypeptides29, Table S2: Frequently prescribed medications with known P450 metabolism which are neither PIMs nor PPOs, Table S3: Foods and lifestyle drugs with known P450 metabolism which are neither PIMs nor PPOs.
