# Supplementary material for: Improving the Care of Older Patients by Decreasing Potentially Inappropriate Medications, Potential Medication Omissions, and Serious Drug Events Using Pharmacogenomic Data about Variability in Metabolizing Many Medications by Seniors
Source: Geriatrics (Basel). 2020 Sep 27;5(4):64. doi: 10.3390/geriatrics5040064 (PMC7709619; doi:10.3390/geriatrics5040064)
Supplement: Supplementary file 1 [file geriatrics-05-00064-s001.pdf]

Table S1. Genetic influences on efflux pumps and organic anion-transporting polypeptides[27]				
**p-glycoprotein efflux pump (p-gp)**			**Organic anion-transporting polypeptides (OATP)**	
**Substrates**	**Inducers**	**Inhibitors**	**Substrates**	**Inhibitors**
Pump removes drugs from cells		Pump prevalent in gut and inhibition prevents pump putting medications back into gut	OATPs are influx pumps and pump drug into cells	If inhibitor acts on kidneys or liver, it increases drug levels. If at gut it decreases drug levels.
Amiodarone, apixaban, citalopram, cyclosporine, dabigatran, dexamethasone, digoxin, diltiazem, edoxaban, erythromycin, loperamide, lovastatin, posaconazole, prednisone, ranitidine, rifampin, rivaroxaban, sertraline, verapamil	Carbamazepine, phenobarbital, dexamethasone, phenytoin, primidone, rifampin, St. John's Wort	Amiodarone, carvedilol, clarithromycin, cyclosporine, diltiazem, dronedarone, duloxetine, erythromycin, grapefruit, itraconazole, ketoconazole, mefloquine, mifepristone, posaconazole, propafenone, quinidine, tamoxifen, ticagrelor, verapamil	Angiotensin II Receptor Blockers (ARBs), ciprofloxacin, erythromycin, montelukast, statins	Clarithromycin, cyclosporine, erythromycin, gemfibrozil, grapefruit, ketoconazole, rifampin

Note: Organic anion transporters (coded by gene SLCO1B1) are on the sinusoidal membranes of hepatocytes and uptake weakly acidic compounds such as statins, methotrexate and bilirubin. Patients homozygous for SLCO1B1*5/*5 or *5/*17 or *15/*17 have a statin area under the curve (AUC) of 221%.

Table S2. Frequently prescribed medications with known P450 metabolism which are neither PIMs nor PPOs								
	P450 cytochrome isoforms							
**Medication**	**1A2**	**2B6**	**2C8**	**2C9**	**2C19**	**2D6**	**2E1**	**3A457**
**Other medications, listed alphabetically**								
Amlodipine								Amlodipine; *3A4 Amlodipine*
Amphetamine						Amphetamine; *Amphetamine*		
Atomoxetine					Atomoxetine	Atomoxetine; *Atomoxetine*		Weak inhibitor
Atorvastatin								Atorvastatin; *3A4 Atorvastatin*
Budesonide								*3A4 Budesonide*
Buprenorphine								*3A4 Buprenorphine*
Bupropion	**Bupropion**					Strong inhibitor		
Chloramphenicol					Chloramphenicol, also inhibitor			Inhibitor
Cimetidine	Weak inhibitor		**Cimetidine**	**Cimetidine**	**Cimetidine** inhibitor	Weak inhibitor		**Cimetidine**; weak inhibitor
Ciprofloxacin	Strong inhibitor							Inhibitor
Cisapride								Cisapride
Cilostazol								Cilostazol

Clarithromycin								Clarithromycin; also strong inhibitor; *3A4 Clarithromycin*
Clobazam				Clobazam				
Clonazepam								*3A4 Clonazepam*
Cotrimoxazole				Weak to moderate inhibitor				
Dextromethorphan						Dextromethorphan; *Dextromethorphan*		Dextromethorphan
Domperidone								Domperidone; *3A4 Domperidone*
Erythromycin								Erythromycin; also moderate inhibitor; *3A4 Erythromycin*
Fenofibrate				Inhibitor				
Finasteride, also **CYP 2A6, 2A7, 2A13, 3A43, 26A1**		**Finasteride**						**Finasteride 3A4,3A5,3A7**; Finasteride
Fluconazole			**Fluconazole**	**Fluconazole** inhibitor	**Fluconazole**			**Fluconazole 3A4** moderate inhibitor
Fluticasone								*3A4 Fluticasone*
Fluvastatin				Fluvastatin, also inhibitor				
Gemfibrozil			Strong inhibitor					

Glibenclamide				Glibenclamide				
Glipizide				Glipizide				
Hydrocodone						*Hydrocodone*		*3A4* Hydrocodone
Insulin	Inducer							
Isoniazid				Inhibitor	Inhibitor		Inducer	
Itraconazole								Strong inhibitor
Ketoconazole					Inhibitor			Strong inhibitor
Methadone	*Methadone*	**Methadone;** Methadone	**Methadone**	*Methadone*	**Methadone;** *Methadone*	**Methadone;** inhibitor		**Methadone 3A4,3A7** Methadone; *3A4 Methadone*
Metronidazole				Inhibitor				
Midazolam								Midazolam; *3A4 Midazolam*
Mifepristone								Inhibitor
Mirtazapine	Mirtazapine					*Mirtazapine*		
Moclobemide					Moclobemide	Inhibitor		
Modafinil	Inducer				inhibitor			Inducer
Nateglinide				Nateglinide				
Nefazodone								Strong inhibitor
Norethindrone, also CYP 1A1								**Norethindrone 3A5**
Norfloxacin, Ofloxacin					inducer			Inhibitor
Ondansetron	Ondansetron; *Ondansetron*					Ondansetron; *Ondansetron*		Ondansetron

Oxcarbazepine		**Oxcarbazepine**	**Oxcarbazepine**		**Oxcarbazepine**			**Oxcarbazepine 3A4, 3A7**
Phenformin						Phenformin		
Pimozide								Pimozide
Probenicid				Inhibitor	Inhibitor			
Proguanil					Proguanil			
Quinidine						Strong inhibitor		Quinidine
Ranitidine						Inhibitor		
Repglinide			Repglinide					
Rifampin	Inducer	Inducer	Inducer	Inducer		Inducer		Inducer
Ritonavir					Inducer	Inhibitor		Ritonavir; strong inhibitor
Saxagliptin								*3A4* Saxagliptin
Selegiline		Selegiline						
Sildenafil				*Sildenafil*				Sildenafil; *3A4 Sildenafil*
Simvastatin								Simvastatin; *3A4 Simvastatin*
Sulfamethoxazole				Inhibitor; *Sulfamethoxazole*				
Tadalfil								*3A4 Tadalfil*
Tamoxifen		**Tamoxifen;**		Tamoxifen; *Tamoxifen*		**Tamoxifen;** Tamoxifen; *Tamoxifen*		**Tamoxifen 3A4, 3A5;** Tamoxifen; *3A4 Tamoxifen*

Telithromycin								Telithromycin; also strong inhibitor
Terbinafine			**Terbinafine**	**Terbinafine**	**Terbinafine**	Strong inhibitor		**Terbinafine 3A4**
Terfenadine								Terfenadine
Tizanidine								
Thioridazine						Thioridazine		
Tolbutamide				Tolbutamide				
Trazodone						*Trazodone*		Trazodone; *3A4 Trazodone*
Triazolam								*3A4 Triazolam*
Trimethoprim			Moderate inhibitor					
Valproic acid **(CYP2A6)**		**Valproic acid**		Valproic acid; **Valproic acid**				
Vardenafil				*Vardenafil*				
Voriconzole		Inhibitor		Voriconzole, also moderate inhibitor	Voriconzole, also moderate inhibitor			Voriconzole also moderate inhibitor
Zolmitriptan	Zolmitriptan							
Zopiclone								*3A4 Zopiclone*

Note: The Flockhart tables define a Moderate inhibitor is one that causes a > 2-fold increase in the plasma AUC values or 50-80% decrease in clearance. A Weak inhibitor is one that causes a > 1.25-fold but < 2-fold increase in the plasma AUC values or a 20-50% decrease in clearance.

**Table S3. Foods and lifestyle drugs with known P450 metabolism which are neither PIMs nor PPOs**								
	**P450 cytochrome isoforms**							
Lifestyle, diet								
**Medication**	**1A2**	**2B6**	**2C8**	**2C9**	**2C19**	**2D6**	**2E1**	**3A457**
Broccoli	Inducer							
Brussels sprouts	Inducer							
Char grilled meat	Inducer							
Cocaine						Inhibitor	**Cocaine**	Cocaine
Caffeine, also CYP2A6	**Caffeine;** Caffeine; *Caffeine*		**Caffeine**	**Caffeine**			**Caffeine**	**Caffeine 3A4**
Ethanol							**Ethanol**; Ethanol, also inducer	
Grapefruit								moderate inhibitor
Tobacco smoking	Inducer							
